# Direct synthesis of oxalic acid via oxidative CO coupling mediated by a dinuclear hydroxycarbonylcobalt(III) complex

**DOI:** 10.1038/s41467-023-38442-4

**Published:** 2023-05-12

**Authors:** Yingzhuang Xu, Songyi Li, Huayi Fang

**Affiliations:** School of Materials Science and Engineering, Tianjin Key Lab for Rare Earth Materials and Applications, Nankai University, Tianjin, 300350 China

**Keywords:** Organometallic chemistry, Organometallic chemistry, Sustainability

## Abstract

Oxidative coupling of CO is a straightforward and economic benign synthetic route for value-added *α*-diketone moiety containing C_2_ or higher carbon compounds in both laboratory and industry, but is still undeveloped to date. In this work, a rare coplanar dinuclear hydroxycarbonylcobalt(III) complex, bearing a Schiff-base macrocyclic equatorial ligand and a *μ-κ*^1^(O):*κ*^1^(O’)-acetate bridging axial ligand, is synthesized and characterized. The Co(III)-COOH bonds in this complex can be feasibly photocleaved, leading to the formation of oxalic acid. Moreover, the light-promoted catalytic direct production of oxalic acid from CO and H_2_O using O_2_ as the oxidant with good selectivity (> 95%) and atom economy at ambient temperature and gas pressure based on this dicobalt(III) complex have been achieved, with a turnover number of 38.5. The ^13^C-labelling and ^18^O-labelling experiments confirm that CO and H_2_O act as the sources of the -COOH groups in the dinuclear hydroxycarbonylcobalt(III) complex and the oxalic acid product.

## Abstract (web summary)

Oxidative coupling of CO is a straightforward but currently underdeveloped economically benign synthetic route to target α-diketones. Here, the authors synthesize a rare coplanar dinuclear hydroxycarbonylcobalt(III) complex and demonstrate that the Co – carboxylate bonds can be easily cleaved leading to the formation of oxalic acid.

## Introduction

**Fig. 1:**
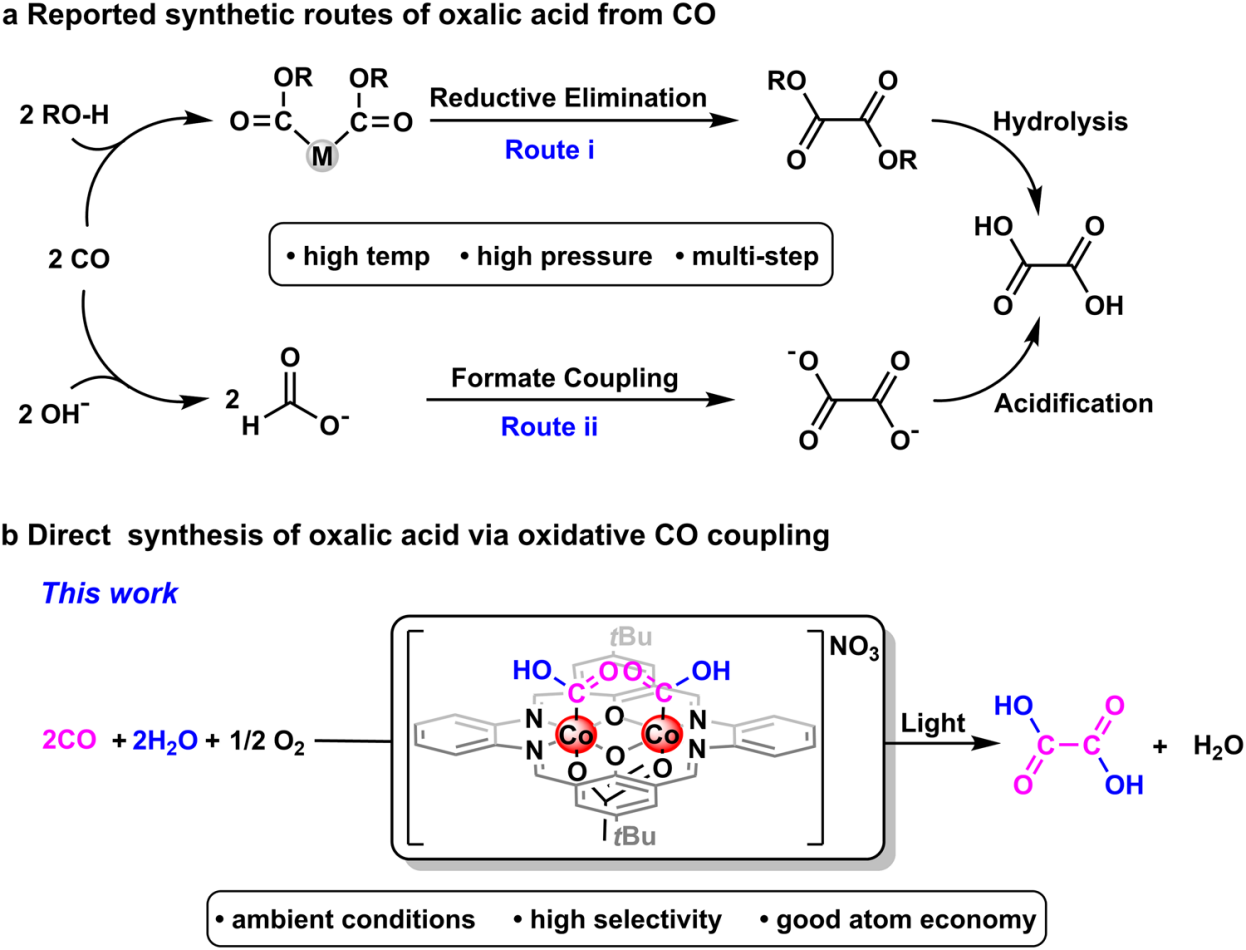
Synthetic routes of oxalic acid from CO. **a** Reported synthetic routes of oxalic acid from CO. **b** Direct synthesis of oxalic acid via oxidative CO coupling.

Selective C-C coupling of carbon monoxide (CO), which is one of the central C_1_ feedstocks in both laboratory and chemical industries^1–3^, is long known as an important and efficient synthetic route for C_2_ and higher carbon products^4–7^. The majority of previously documented CO coupling strategies could be sorted into two categories as oxidative CO coupling and reductive CO coupling. Researches on reductive CO coupling can be traced back to the early nineteenth century during which molten potassium is reported to reductively couple CO to form the [C_2_O_2_]_*n*_^2*n*-^ anions^8,9^. Since then, a series of *s*-,^7^ *p*-,^10,11^ *d*-,^12–21^ and *f*-block^22–24^ element complexes have been demonstrated to be capable for the reductive CO coupling, affording a number of C-C bond formation products including ynediolates, enediolates, and oxygen-free hydrocarbons. In comparison, the research of oxidative CO coupling is much lagged behind and only a limited number of examples have been reported till now^25^. For instances, organolithiums are known to react with CO to afford 1,2-diketones as the oxidative CO coupling products^26–28^. Besides, some transition metal complexes, such as Re(I/III) and Pd(II) complexes, are also illustrated to be capable of mediating both stoichiometric and catalytic CO oxidative couplings^29–35^ (Fig. 1a, route i). High pressure of CO (65−80 atm) is noted to be crucial for good yields and selectivity of the reported catalytic systems, and carbonates are usually found to form as major products at low CO pressure in this processes34. Mechanistically, the high gas pressure is a prerequisite for the single site pathway, in which two CO molecules are activated at one metal center, that governs the aforementioned processes. Therefore, the cooperative mechanism standing on two or more geometrically correlated reacting sites potentially paves the way for oxidative CO coupling at ambient conditions^36^.

Oxalic acid plays indispensable roles in various industry processes, such as metal processing, rare earth extraction, leather treatment and pharmaceutical, with an annual market of 350,000 tons^37^. And CO is one of the major raw materials used in the applied commercially viable production of oxalic acid, wherein CO is firstly converted to the alkali formate intermediate and the oxalic acid is obtained by the following formate coupling and acidification (Fig. 1a, route ii). However, harsh conditions, including high reaction temperature and CO pressure, are necessary for this multistep time- and energy-consuming route. Meanwhile, the formation of carbonates byproducts is inevitable as a consequence of the decompositions of the alkali formates and oxalate product. In principle, oxalic acid can be straightforwardly prepared by the oxidative coupling of CO with H_2_O, while to the best of our knowledge, no such process has been reported yet.

Here, we show the light-promoted direct and selective production of oxalic acid from CO and H_2_O with good atom economy mediated by a coplanar dinuclear hydroxycarbonylcobalt(III) complex using O_2_ as the oxidant under ambient reaction temperature (room temperature) and gas pressure (1 atm of CO and 1 atm of O_2_) with a turnover number of 38.5.

## Results

### Synthesis and characterizations of dicobalt complexes 1-5

**Fig. 2:**
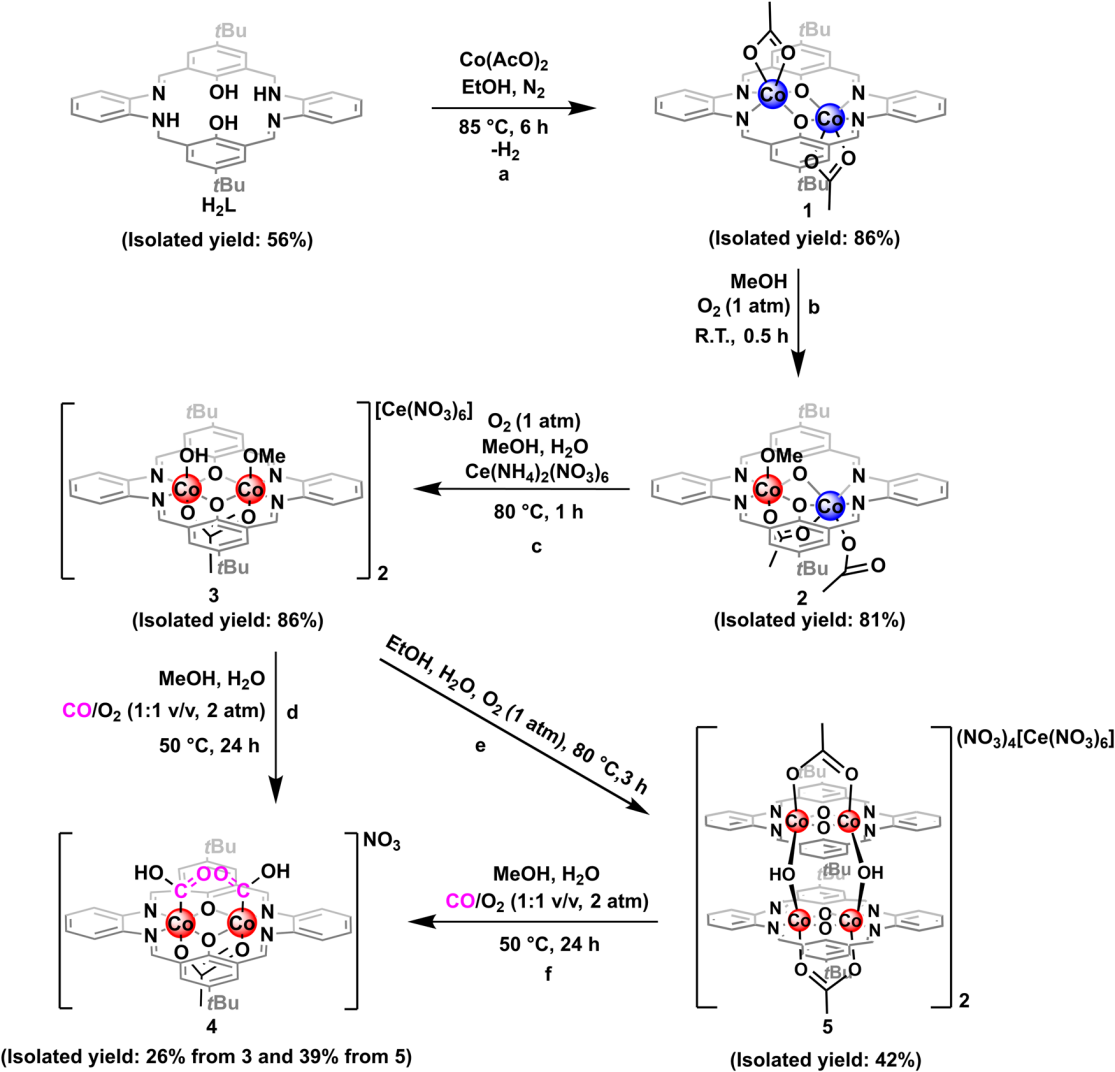
Procedures for the synthesis of key complexes. **a** Synthesis of **1**. **b** Synthesis of **2**. **c** Synthesis of **3**. **d** Synthesis of **4** from **3**. **e** Synthesis of **5**. **f** Synthesis of **4** from **5**.

**Fig. 3:**
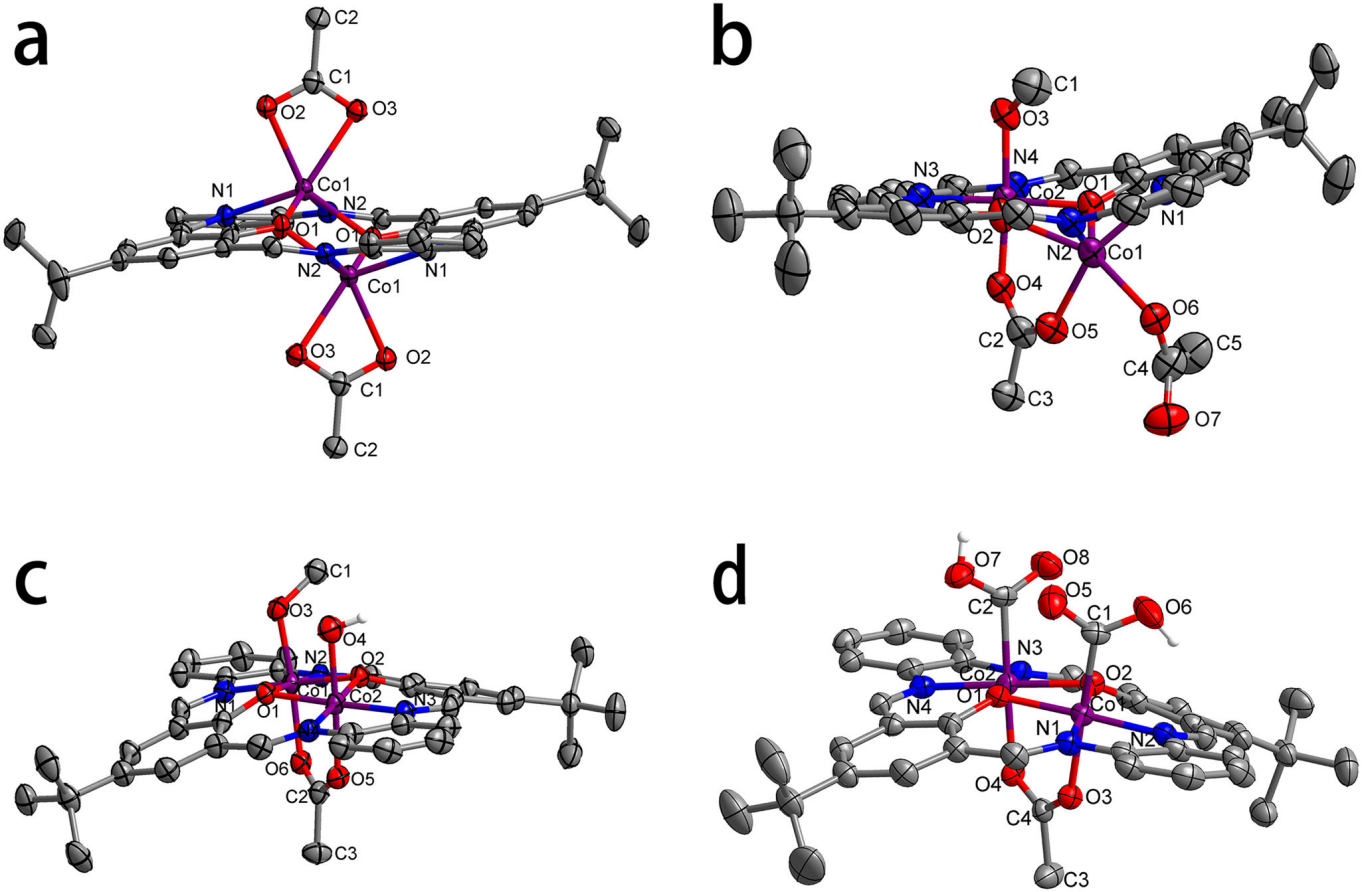
ORTEP representations (50% probability) of the X-ray structures. **a** For **1**. **b** For **2**. **c** For the cationic part of **3**. **d** For the cationic part of **4**. Crystallized solvent molecules, counteranions, and hydrogen atoms, except for those on oxygen atoms, have been omitted for clarity. For details about these X-ray structures see Supplementary Data 1.

The macrocyclic ligand (**H**_**2**_**L**) having two coplanar metal binding sites is synthesized based on the previously reported method^38^. The reaction of **H**_**2**_**L** with 2 equivalents of Co(OAc)_2_ under N_2_ in ethanol followed by recrystallization with Et_2_O gives the isolation of yellow complex **1** in 86% yield (Fig. 2a). The solid-state structure of **1** shows that the two Co(II) centers sit at different sides of the ligand, with extra coordinations from acetate ligands (Fig. 3a). The two secondary benzylamine moieties in the ligand framework are converted to imines during the metalation, probably via the cobalt mediated dehydrogenation^39^. The ^1^H NMR monitoring of the synthesis of **1** in a J. Young NMR tube showed a singlet resonance at *δ* = 4.35 ppm, indicating the formation of H_2_ (Supplementary Fig. 1). The effective magnetic moment (7.67 *μ*_B_) measured for **1** at room temperature is indicative of the presence of two high-spin (*S* = 3/2) Co(II) centers (Supplementary Fig. 2). The spin-orbital splitting energy (15.9 eV) obtained from X-ray photoelectron spectroscopy (*XPS*) measurement is also in agreement with the oxidation state assignments of +2 for the two cobalt centers (Supplementary Fig. 3). **1** can be readily oxidized by O_2_ in methanol at room temperature to provide a red mixed-valent dicobalt(II/III) complex **2**, as supported by the *XPS* measurement (the measured spin-orbital splitting energies for Co(II) and Co(III) are 15.6 eV and 14.8 eV, respectively, Supplementary Fig. 5), in 81% isolated yield (Fig. 2b). The solid state structure of **2** shows that the Co(III) center well accommodates in the N_2_O_2_ plane and occupies an octahedral coordination geometry, while the larger Co(II) center still sits above the ligand framework (Fig. 3b). In accordance, the Co(III)-O_equatorial_ (1.881(3)/1.868(2) Å) and Co(III)-N_equatorial_ (1.867(3)/1.870(3) Å) bonds in **2** are significantly shorter than those in **1** (Co(II)-O_equatorial_: 2.063(2)/2.144(3) Å; Co(II)-N_equatorial_: 2.108(3)/2.071(3) Å). The effective magnetic moment (4.12 *μ*_B_) of **2** indicates a high spin (*S* = 3/2) Co(II) center and a low spin (*S* = 0) Co(III) center (Supplementary Fig. 6).

After the addition of 1.1 equivalent of Ce(NH_4_)_2_(NO_3_)_6_, the brown dicobalt(III) complex **3** is obtained via the further oxidation of **2** at elevated reaction temperature in an isolated yield of 86% (Fig. 2c). The measured spin-orbital splitting energy (15.1 eV) is in line with the presence of only Co(III) centers (Supplementary Fig. 7). ^1^H NMR spectrum of **3** displays sharp resonances in the range of *δ* = 1 – 10 ppm, showing the diamagnetic feature of **3** (Supplementary Fig. 10). As shown in Fig. 3c, both of the Co(III) centers in **3** well fit into the N_2_O_2_ plane of the ligand. The Co(III)-O_equatorial_ (1.914(2)/1.894(2)/1.896(2)/1.900(2) Å) and Co(III)-N_equatorial_ (1.859(3)/1.874(3)/1.868(3)/1.866(3) Å) bond lengths are very comparable to those for the Co(III) center in **2**. The two Co(III) centers are bridged by an acetate ligand in a *μ*-*κ*^1^(O):*κ*^1^(O’) manner, while the hydroxyl and methoxyl axial ligands are coordinated to each of the Co(III) centers from the other side.

**Fig. 4:**
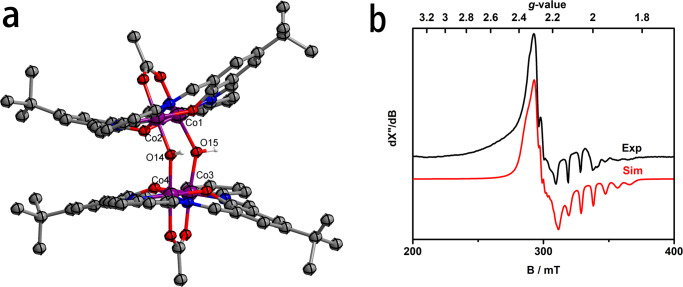
Characterizations of 5. **a** ORTEP representation (50% probability) of the cationic part of **5**. Crystallized solvent molecules, counteranions, and hydrogen atoms, except for those on oxygen atoms, have been omitted for clarity. For details about this X-ray structure see Supplementary Data 1. **b** Experimental (black) and simulated (red) X-band EPR spectrum of **5** in solid state.

Treatment of **3** in methanol (with the presence of 0.01% *w/w* of water) with 1 atm of CO at 50 °C produces the diamagnetic dinuclear hydroxycarbonylcobalt(III) complex (**4**) within 24 h (Fig. 2d). The spin-orbital splitting energy (15.0 eV) derived from *XPS* measurement is nearly identical to that of **3** (Supplementary Fig. 12). Besides the different axial ligands, the molecular structure of **4** largely resemble the geometrical features of **3** (Fig. 3d). The C = O (1.193(5)/1.207(4) Å) and C(O)-OH (1.231(5)/1.249(4) Å) bond lengths are in the normal range reported for other hydroxylcarbonyl metal complexes^40,41^. The IR spectrum of **4** shows two close absorptions at 1697 and 1670 cm^−1^, in satisfactory agreement with the modelling results based on density functional theory (DFT) calculations (1724 and 1685 cm^−1^). When ^13^CO is used in the synthesis of **4**, the two absorptions are red-shifted to 1662 and 1651 cm^−1^ (Supplementary Fig. 13, the calculated values are 1685 and 1648 cm^−1^). ^1^H NMR signals of the two carboxylic protons are found at *δ* = 13.34 and 13.08 ppm, respectively (Supplementary Fig. 14). In addition, the thermogravimetric analysis of **4** is performed and a weight loss (10.1 *wt*%) at 280−290 °C, as a result of the release of two -COOH groups, is observed (Supplementary Fig. 17). **4** is envisioned to form via the CO insertion into the Co(III)-OH bonds of the proposed dinuclear hydroxocobalt(III) intermediate, which could be generated via the reaction of **3** with H_2_O that results in the replacement of axial methoxyl ligand to hydroxyl ligand. Although attempts for the isolation of this intermediate are unsuccessful, tetranuclear cobalt complex **5** is obtained in 42% isolated yield by heating the ethanol solution of **3** at 80 °C for 3 hours as shown in Fig. 2e. The measured spin-orbital splitting energy of cobalt centers in **5** (15.0 eV) is nearly identical to those of Co(III) centers in **3** and **4** (Supplementary Fig. 18). The lengths of the Co-O_*μ*-OH_ bonds (1.905(4)/1.909(4)/1.907(4)/1.895(4) Å, Fig. 4a) in **5** are very comparable to the reported values (1.888 − 1.912 Å) for dinuclear cobalt(III) *µ*-hydroxo species^40,41^, but is shorter than the typical Co(III)-OH_2_ bond (1.945 Å)^42,43^ and significantly longer than the Co(III)-O_*μ*-O_ bonds (1.783−1.796 Å)^44^. The effective magnetic moment (1.14 *μ*_B_) measured for **5** at room temperature is indicative of the presence of only one unpaired electron (Supplementary Fig. 19). The unrestricted corresponding orbital analysis and the calculated spin density of **5** show that the unpaired electron density is on the cobalt centers (Supplementary Fig. 20). The EPR measurement of **5** in solid state is also conducted at 97 K. An anisotropic signal (*g*_1_ = 2.023, *g*_2_ = 2.222, *g*_3_ = 2.305) with well resolved hyperfine splitting from Co nucleus (*I* = 7/2, *A*_1_ = 264.00 MHz, *A*_2_ = 64.50 MHz, *A*_3_ = 60.00 MHz) is observed (Fig. 4b). Furthermore, **4** can be obtained by the reaction of **5** with CO and O_2_ (Fig. 2f). A plausible formation pathway of **5** from **3** via the proposed dinuclear hydroxylcobalt(III) intermediate is depicted in Supplementary Fig. 21.

### Light promoted production of oxalic acid catalyzed by 4

The relatively short distance (3.419 Å) between the two carbon atoms of the -COOH ligands in **4** encourages us to further examine the production of oxalic acid from **4**. Irradiation of **4** using Xe-lamp as the light source is carried out under N_2_ at room temperature, and oxalic acid is observed to form in 57% yield. By replacing the N_2_ atmosphere to CO/O_2_ mixture gas (1:1 *v/v*, 2 atm), the catalytic production of oxalic acid with good selectivity (> 95%) is achieved with a turnover number (TON) of 38.5 (Table 1). Only trace amount (TON ~ 0.3) of dimethyl carbonate, which is a commonly seen side product during the Pd(II) complexes catalyzed production of oxalic acid^31,34,35^, is formed, and, meanwhile, no dimethyl oxalate is found in this process. It is worth noting that the formation of H_2_O_2_ (TON ~ 0.1) is also detected, which might indicate the intermediacy of the dinuclear hydroxocobalt(III) complex in the catalytic cycle. It is noteworthy that yellow solid was observed to precipitated out of the reaction solution during the catalytic production of oxalic acid as a consequence of the catalyst decomposition. *XPS* measurement of this precipitate shows the presence of unidentified Co(II) complexes (Supplementary Fig. 23). Of note, both **3** and **5** are also capable for catalyzing the production of oxalic acid under same conditions, but in lower efficiency with TONs of 10.4 and 11.3, respectively, while **1** and **2** are not suitable candidates for this catalysis (Table 1). To further confirm the origins of the carbonyl and hydroxyl groups in the oxalic acid product, isotope labelling experiments have been conducted. In ^13^C-labelling experiment, the produced oxalic acid is converted to calcium oxalate by reacting with CaCl_2_ and then collected for IR measurements. When ^13^CO is used, the recorded C = O, C(O)-OH and (O)C-C(O) bond stretching frequencies of the obtained calcium oxalate are all red-shifted, confirming the formation of Ca^13^C_2_O_4_ (Supplementary Fig. 24). Similar shifting trend has also been reported for ^13^C-labeled and unlabeled oxalates of sodium and potassium^45,46^. For the ^18^O-labelling experiment in which H_2_^18^O and ^16^O_2_ are used, only the ^18^OH labelled oxalic acid is observed by MS measurement (*m/z*^-^ = 93.0 [M-H]^-^, Supplementary Fig. 25b). In comparison, no ^18^OH labeling product is seen when H_2_^16^O and ^18^O_2_ were submitted to the catalytic system (Supplementary Fig. 25c). These results support that H_2_O rather than O_2_ is the source of the hydroxyl group in the produced oxalic acid.

**Table. 1:** The efficiency and selectivity of different catalysts for oxalic acid production

TONs							
Catalyst						CO_2_	H_2_O_2_
**1**	n.d.	n.d.	0.2	n.d.	trace	n.d.	n.d.
**2**	0.4	n.d.	0.2	n.d.	trace	n.d.	n.d.
**3**	10.4	n.d.	1.3	1.4	trace	n.d.	0.9
**4**	38.5	n.d.	0.3	2.4	0.6	n.d.	0.1
**5**	11.3	n.d.	trace	2.7	trace	n.d.	0.1

All the catalytic reactions were carried out in methanol under 2 atm of CO/O_2_ mixture gas (1:1 *v/v*) at room temperature under Xe-lamp irradiation for a reaction time of 28 hours; TONs were determined by ^1^H NMR (for formic acid), gas chromatograph (for dimethyl oxalate, dimethyl carbonate, methyl formate and CO_2_), liquid chromatograph (for oxalic acid), and chemical analysis (iodometric method, Neocuproine/CuSO_4_ titration method and Ce(SO_4_)_2_ titration method for H_2_O_2_); *n.d.* not detected.

### Computational studies on the production of oxalic acid

**Fig. 5:**
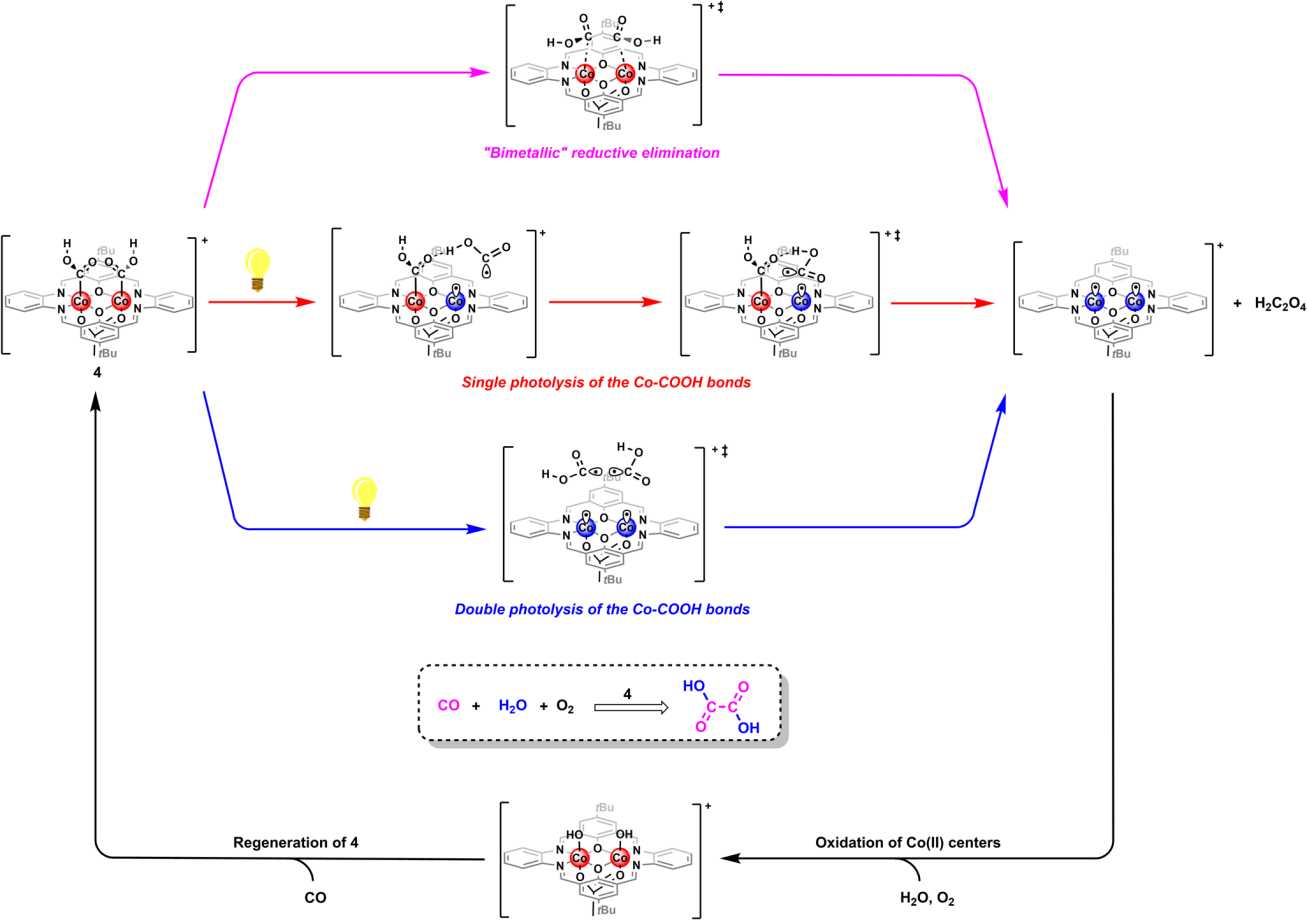
Plausible mechanism for the production of oxalic acid catalyzed by 4. The counteranions have been omitted for clarity.

**Fig. 6:**
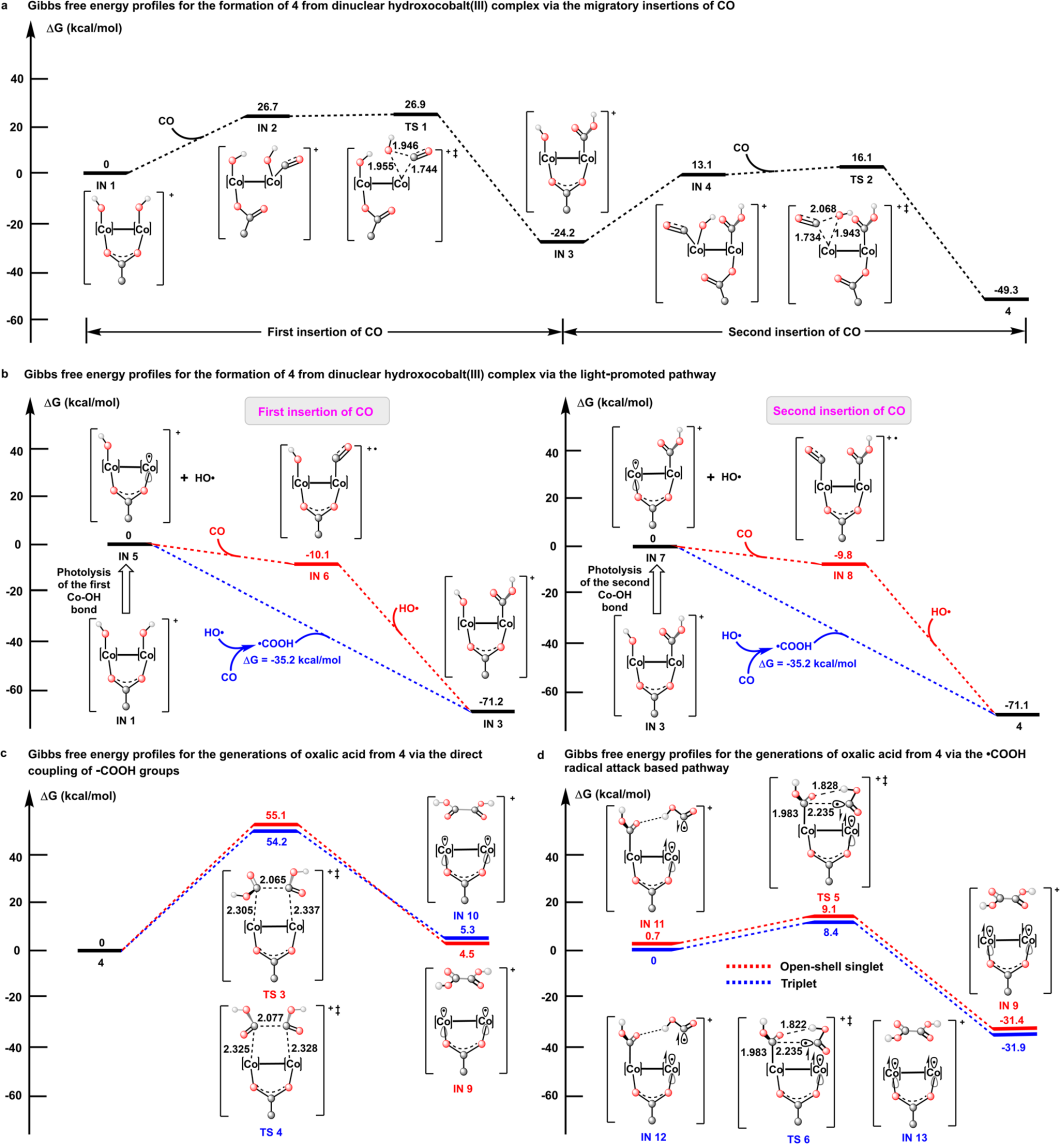
Mechanistic investigations. Gibbs free energy profiles for the formation of 4 from dinuclear hydroxocobalt(III) complex via: a The classical migratory insertions of CO; **b** The light-promoted pathway. Gibbs free energy profiles for the generations of oxalic acid from **4** via: **c** The direct coupling of -COOH groups; **d** The hydroxycarbonyl radical attack based pathway. Bond lengths and distances are provided in Å, detailed structures of the intermediates and transition states have been summarized in Supplementary Data 2. The counteranions have been omitted for clarity.

Given all the aforementioned results, the plausible mechanism for the oxalic acid production catalyzed by **4** is shown in Fig. 5. In addition, computational studies of all these proposed pathways based on DFT calculations have been conducted using ORCA program package^47,48^ (Fig. 6). The classical migratory CO insertions into the Co(III)-OH bonds in the dinuclear hydroxocobalt(III) intermediate is principally a plausible pathway for the generation of **4**. The calculated penitential energy surface (PES) of the migratory insertion-based pathway is shown in Fig. 6a. The coordination of CO on the Co(III) center in **IN 1** is significantly endothermic with a Gibbs free energy change of 26.7 kcal/mol. The -COOH group is formed with a Gibbs free energy change (ΔG) of − 50.9 kcal/mol via the succeeding migratory CO insertion, of which a very low activation energy (E_a_ = 0.2 kcal/mol) is calculated. For the coordination of CO to the Co(III) center in **IN 3**, the Gibbs free energy raises by 37.3 kcal/mol. Although the following migratory CO insertion only needs to traverse a small activation energy (E_a_ = 3.0 kcal/mol) and is exothermic (ΔG = − 62.4 kcal/mol), the whole pathway is not likely to occur at ambient reaction temperature as a result of the involvements of the energetically unfavored CO coordination processes. As an alternative, a light promoted pathway for the generation of **4**, in which the Co(III)-OH bonds are photocleaved prior to the reaction with CO, is proposed (Supplementary Fig. 30 and Fig. 6b). For the first CO insertion, the coordination of CO to the Co(II) center in **IN 5** is moderately exothermic (ΔG = − 10.1 kcal/mol) and the succeeding hydroxyl radical attack on the coordinated CO that completing the formation of the first -COOH group is strongly energetically favored (ΔG = − 61.1 kcal/mol). It is worth noting that the coupling of Co(II) center with hydroxycarbonyl radical, which is formed via the feasible reaction of hydroxyl radical with CO (ΔG = −35.2 kcal/mol), is also a viable pathway for the CO insertion. A very similar PES is calculated for the second CO insertion. The calculated PESs are supportive of the smooth proceeding of the formation of **4** via the light-promoted pathway.

For the production of oxalic acid from **4**, three different pathways (Fig. 5) can be envisioned as: (i) direct -COOH group coupling in a “bimetallic” reduction elimination manner; (ii) one of Co(III)-COOH bonds in **4** is photocleaved and the succeeding attack on the intact Co(III)-COOH bond by the hydroxycarbonyl radical that yields the oxalic acid; and (iii) the photocleavage occurs for both of the Co(III)-COOH bonds in **4** and oxalic acid forms by coupling of the produced free hydroxycarbonyl radicals. The first two pathways are further investigated computationally. Very high activation energies (E_a_ = 55.1 kcal/mol for the formation of *s*-*E*-oxalic acid and E_a_ = 54.2 kcal/mol for the formation of *s*-*Z*-oxalic acid) are found for the direct coupling of -COOH group thus excluding their occurrences at ambient reaction temperature (Fig. 6c). For the hydroxycarbonyl radical attack based pathway, the calculated Gibbs free energy profiles show that all the radical attack processes with different multiplicities (*S* = 0 and 1) and hydrogen bonding patterns (O-H^…^O = C-OH and O-H^…^(OH)C = O, which lead to the formation of *s*-*E*-oxalic acid and *s*-*Z*-oxalic acid, respectively) only need to go across small energy barriers (7−9 kcal/mol) and are strongly energetically favored with large Gibbs free energy change of 32−34 kcal/mol (Fig. 6d and Supplementary Fig. 31). Therefore, the two light promoted pathways, initiated by single and double photocleavage of the Co(III)-COOH bonds in **4**, are both regarded to be responsible for the presented production of oxalic acid catalyzed by **4**.

## Discussion

In summary, a series of dicobalt complexes (**1-4**) bearing planar macrocyclic ligands have been synthesized and characterized. The light irradiation of the rare coplanar dinuclear hydroxycarbonylcobalt(III) complex (**4**) leads to the photocleavage of the Co(III)-COOH bonds and the formation of oxalic acid. Inspired by this result, a strategy for the direct and selective production of oxalic acid with good atom economy via oxidative CO coupling at ambient conditions mediated by **4** is developed. The ^13^C- and ^18^O-labelling experiments confirm that CO and H_2_O are the sources of the -COOH groups in **4** and oxalic acid product. The presented results may provide the basis for developing new strategy for CO upgrading, and shed light in designing of bimetallic complex platforms with novel reactivity. Further explorations on extending the reactivity scope of this bimetallic system are underway.

## Methods

All manipulations involving air-sensitive materials were performed under N_2_ atmosphere using standard Schlenk techniques or in gloveboxes. Chemicals were purchased from Sigma-Aldrich, Alfa Aesar, J&K Scientific Ltd. or Cambridge Isotope Laboratory Inc. All chemicals were used without further treatment.

### Synthesis of ligand and dicobalt complexes

#### H_2_L

To a stirred solution of 5-*t*-butyl-2-hydroxyisophthalaldehyde (0.7828 g, 3.800 mmol) in methanol (40.0 mL) containing AcOH (1840 μL, 31.04 mmol) was added a solution of *o*-phenylenediamine (0.4104 g, 3.800 mmol) in methanol (40.0 mL) dropwise for 30 min at room temperature and then stirred for 6 h. The methanol solution was then removed by filtration, and the residual solid was collected, followed by the removal of all volatiles under vacuum, giving the title ligand as a yellow powder (56% yield). ^**1**^**H NMR** (400 MHz, CDCl_3_) *δ* (ppm): 13.55 (s, 2H, O*H*), 8.63 (s, 2H, NC*H*), 7.42 (s, 1H, Ar*H*), 7.42 (s, 1H, Ar*H*),7.35 (s, 1H, Ar*H*), 7.34 (s, 1H, Ar*H*), 7.07 (d, *J* = 7.72 Hz, 1H, Ar*H*), 7.05 (d, *J* = 7.72 Hz, 1H, Ar*H*), 6.98 (br, 1H, Ar*H*), 6.96 (br, 1H, Ar*H*), 6.79 (t, *J* = 7.56 Hz, 1H, Ar*H*), 6.78 (t, *J* = 7.44 Hz, 1H, Ar*H*), 6.32 (t, *J* = 5.60 Hz, 2H, Ar*H*), 4.46 (s, 2H, Ar*H*), 4.45 (s, 2H, Ar*H*), 3.50 (s, 1H, CN*H*), 3.49 (s, 1H, CN*H*), 1.33 (s, 18H, *t*Bu*H*).

#### (L´)(AcO)_2_Co(II)_2_ (1)

In a nitrogen-filled glovebox, H_2_L (0.0589 g, 0.105 mmol) was dissolved in 8.0 mL of ethanol and followed by the addition of Co(OAc)_2_·4H_2_O (0.0549 g, 0.220 mmol). The reaction mixture was stirred for 6 h at 85 °C, during which time the solution developed from yellow suspension to brown solution. The obtained solution was concentrated to 1.0 mL under reduced pressure, and diethyl ether (30.0 mL) was added. The precipitate was collected by filtration and then dried under vacuum to afford **1** in 86% yield. Crystals suitable for X-ray diffraction were grown by slow evaporation of ethanol solution of **1** at 1 °C for 24 h. **UV/Vis** (ethanol): 304 nm (*ε* = 5.53 × 10^3 ^L mol^−1^ cm^−1^), 314 nm (*ε* = 5.54 × 10^3 ^L mol^−1^ cm^−1^), 331 nm (*ε* = 3.61 × 10^3 ^L mol^−1^ cm^−1^), 412 nm (*ε* = 2.69 × 10^3 ^L mol^−1^ cm^−1^). **Analysis** (calcd., found for C_40_H_40_Co_2_N_4_O_6_·3H_2_O): C (56.88, 56.81), H (5.49, 5.51), N (6.63, 6.43).

#### (L´)(AcO)_2_(MeO)Co(II)Co(III) (2)

Complex **1** (0.0844 g, 0.100 mmol) was charged into a 25.0 mL Schlenk flask, followed by the addition of methanol (6.0 mL) at room temperature. After three freeze-pump-thaw cycles, 1 atm of O_2_ was inflated into the Schlenk flask. The solution was then stirred for 30 min, yielding a red solution. The obtained solution was concentrated to 1.0 mL under reduced pressure, and diethyl ether (10.0 mL) was added. The orange precipitate was collected by filtration and then dried under vacuum to afford **2** in 81% yield. X-ray quality crystals of **2** could be obtained from a concentrated methanol solution over three days at room temperature. **UV/Vis** (ethanol): 298 nm (*ε* = 7.43 × 10^3 ^L mol^−1^ cm^−1^), 418 nm (*ε* = 2.13 × 10^3 ^L mol^−1^ cm^−1^), 474 nm (*ε* = 1.25 × 10^3 ^L mol^−1^ cm^−1^). **Analysis** (calcd., found for C_41_H_44_Co_2_N_4_O_7_·H_2_O): C (58.58, 58.13), H (5.52, 5.46), N (6.66, 6.39).

#### [(L´)(AcO)(MeO)(OH)Co(III)_2_]_2_[Ce(NO_3_)_6_] (3)

To a solution of complex **2** (0.1210 g, 0.1440 mmol) in 8.0 mL methanol was added Ce(NH_4_)_2_(NO_3_)_6_ (0.0868 g, 0.158 mmol). After three freeze-pump-thaw cycles, 1 atm of O_2_ was inflated into a 25.0 mL Schlenk flask. The reaction mixture was stirred for 1 h at 80 °C resulting in a color change from red to brown. The solution was concentrated to 4.0 mL under reduced pressure, and diethyl ether (10.0 mL) was added. The brown precipitate was collected by filtration and then dried under vacuum to afforded **3** in 86% yield. X-ray quality crystals of **3** could be obtained from a concentrated methanol solution over three days at room temperature. **UV/Vis** (ethanol): 300 nm (*ε* = 7.02 × 10^3 ^L mol^−1^ cm^−1^), 331 nm (*ε* = 7.58 × 10^3 ^L mol^−1^ cm^−1^), 438 nm (*ε* = 1.60 × 10^3 ^L mol^−1^ cm^−1^). ^**1**^**H NMR** (400 MHz, CD_3_OD) *δ* (ppm): 9.22 (s, 2H, NC*H*), 9.12 (br, 2H, NC*H*), 8.50–8.47 (m, 4H, Ar*H*), 8.34–8.32 (m, 4H, Ar*H*), 7.79–7.72 (m, 4H, Ar*H*), 1.66 (s, 3H, C*H*_3_COO), 1.47–1.46 (br, 18H, *t*Bu*H*). **IR** (potassium bromide disk technique): ν(O-H) = 3425 cm^−1^. **Analysis** (calcd., found for C_78_H_82_CeCo_4_N_14_O_30_·H_2_O): C (44.84, 44.99), H (4.05, 4.13), N (9.39, 9.82).

#### [(L´)(AcO)(COOH)_2_Co(III)_2_][NO_3_] (4)

This complex could be synthesized by two different methods. Method A: In a 25.0 mL Schlenk flask, a solution of complex **3** (0.0350 g, 0.0170 mmol) in 4.0 mL methanol was degassed by three freeze-pump-thaw cycles. 1 atm of O_2_ was inflated into the Schlenk flask, followed by the addition of 1 atm of CO. The solution was stirred for 24 h at 50 °C. The red precipitate was collected by filtration to give **4** as an analytically pure dark red powder in 26% yield. Crystals suitable for X-ray diffraction were obtained by slow evaporation of methanol solution of **4** at room temperature. Method B: In a 25.0 mL Schlenk flask, a solution of complex **5** (0.0320 g, 0.00800 mmol) in 4.0 mL methanol was degassed by three freeze-pump-thaw cycles. 1 atm of O_2_ was inflated into the Schlenk flask, followed by the addition of 1 atm of CO. The solution was stirred for 24 h at 50 °C. The red precipitate was collected by filtration to give **4** as an analytically pure dark red powder in 39% yield. Crystals suitable for X-ray diffraction were obtained by slow evaporation of methanol solution of **4** at room temperature. **UV/Vis** (ethanol): 304 nm (*ε* = 6.73 × 10^3 ^L mol^−1^ cm^−1^), 335 nm (*ε* = 6.21 × 10^3 ^L mol^−1^ cm^−1^), 354 nm (*ε* = 5.74 × 10^3 ^L mol^−1^ cm^−1^), 369 nm (*ε* = 4.95 × 10^3 ^L mol^−1^ cm^−1^), 445 nm (*ε* = 1.76 × 10^3 ^L mol^−1^ cm^−1^). **IR** (potassium bromide disk technique): *ν*(C = O) = 1697 and 1670 cm^−1^; *ν*(^13^C = O) = 1662 and 1651 cm^−1^. ^**1**^**H NMR** (400 MHz, DMSO) *δ* (ppm): 13.34 (s, 1H, COO*H*), 13.08 (s, 1H, COO*H*), 9.49 (s, 4H, NC*H*), 8.46 (br, 8H, Ar*H*), 7.78 (s, 4H, Ar*H*), 1.60 (s, 3H, C*H*_3_COO), 1.45 (br, 18H, *t*Bu*H*). ^**13**^**C NMR** (400 MHz, DMSO) *δ* (ppm) for **4-**^**13**^**C**: 172.12 (s, -COOH). **Analysis** (calcd., found for C_40_H_40_Co_2_N_5_O_11_·H_2_O): C (53.28, 53.49), H (4.58, 4.67), N (7.77, 7.55).

#### [(L´)_2_(AcO)_2_(OH)_2_Co_4_(III)]_2_[NO_3_]_4_[Ce(NO_3_)_6_] (5)

In a 25.0 mL Schlenk flask, a solution of complex **3** (0.0299 *g*, 0.0330 mmol) in 8.0 mL ethanol was degassed by freeze-pump-thaw cycles. One atomsphere of O_2_ was inflated into the Schlenk flask. The solution was stirred for 6 h at 80 °C. The precipitate was filtered off and the filtrate was dried under a vacuum. The residue was washed with cold diethyl ether (−20 °C) to give a brown powder of **5** in 42% yield. Crystals suitable for X-ray diffraction were obtained by slow evaporation of ethanol solution of **5** at room temperature. **UV/Vis** (ethanol): 300 nm (*ε* = 6.47 × 10^3 ^L mol^−1^ cm^−1^), 324 nm (*ε* = 6.26 × 10^3 ^L mol^−1^ cm^−1^), 442 nm (*ε* = 1.41 × 10^3 ^L mol^−1^ cm^−1^). **IR** (potassium bromide disk technique): *ν(*O-H) = 3379 and 3209 cm^−1^. **Analysis** (calcd., found for C_152_H_152_CeCo_8_N_26_O_50_·H_2_O): C (48.39, 47.98), H (4.09, 4.26), N (9.66, 9.58).

#### General procedure for the catalytic production of oxalic acid

A total of 4.0 mL of a methanol solution containing 0.0170 mmol of catalysts (complexes **1**–**5**) was transferred into a 25.0 mL Schlenk flask. After three freeze-pump-thaw cycles, 1 atm of O_2_ was inflated into the Schlenk flask, followed by the addition of 1 atm of CO. The Schlenk flask was set 20.0 cm aside from a 500 W xenon lamp at 30 °C for 28 h. The precipitate was filtered off after the reaction and the filtrate was analyzed by LCMS.

#### Computational details

All calculations were performed on the ORCA quantum chemistry program package (version 5.0.3) with the B97-3c calculation setup^49^. This setup is based on the B97 GGA functional and includes D3 with a three-body contribution and a short-range bond length correction. The modified, stripped-down triple-*ζ* basis, def2-mTZVP^50^ is used in the setup. For the relatively large tetracobalt complex **5**, the crystal structure with the optimized positions for hydrogen atoms was used for the analysis of unrestricted corresponding orbitals and spin density.

## Supplementary information

Supplementary Information

Peer Review File

Description of Additional Supplementary Files

Supplementary Data 1

Supplementary Data 2

## Data Availability

The data associated with this study are available within the article, supplementary information and Supplementary Data. 1 (CIFs for the X-ray structures) and 2 (coordinates of the optimized structures used in computational studies). Crystallographic data for the structures reported in this Article have been deposited at the Cambridge Crystallographic Data Centre, under deposition numbers CCDC 2209326, 2209329, 2209330, 2209332 and 2209333. Copies of the data can be obtained free of charge via https://www.ccdc.cam.ac.uk/structures/. All data are available from the corresponding author.

## References

[1] Braunschweig H (2013). Metal-free binding and coupling of carbon monoxide at a boron–boron triple bond. Nat. Chem..

[2] Jouny M (2019). Formation of carbon–nitrogen bonds in carbon monoxide electrolysis. Nat. Chem..

[3] Sharpe HR (2018). Selective reduction and homologation of carbon monoxide by organometallic iron complexes. Nat. Commun..

[4] Evans WJ, Grate JW, Hughes LA, Zhang H, Atwood JL (1985). Reductive homologation of CO to a ketenecarboxylate by a low-valent organolanthanide complex: synthesis and X-ray crystal structure of [(C_5_Me_5_)_4_Sm_2_(O_2_CCCO)(THF)]_2_. J. Am. Chem. Soc.

[5] Knobloch D, Lobkovsky E, Chirik P (2010). Dinitrogen cleavage and functionalization by carbon monoxide promoted by a hafnium complex. Nat. Chem..

[6] Buss JA (2019). CO coupling chemistry of a terminal Mo carbide: sequential addition of proton, hydride, and CO releases ethenone. J. Am. Chem. Soc..

[7] Liu H-Y (2021). Reductive dimerization of CO by a Na/Mg(I) diamide. J. Am. Chem. Soc..

[8] Wayland B, Fu X (2006). Building molecules with carbon monoxide reductive coupling. Science.

[9] Liebig J (1834). Ueber das verhalten des kohlenoxyds zu kalium. Ann. Chem. Pharm..

[10] Dobrovetsky R, Stephan DW (2013). Stoichiometric metal-free reduction of CO in syn-gas. J. Am. Chem. Soc..

[11] Protchenko AV (2019). Reduction of carbon oxides by an acyclic silylene: reductive coupling of CO. *Angew. Chem*. Int. Ed..

[12] Hu S, Shima T, Hou Z (2020). Hydrodeoxygenative cyclotetramerization of carbon monoxide by a trinuclear titanium polyhydride complex. J. Am. Chem. Soc..

[13] Buss JA, Agapie T (2016). Mechanism of molybdenum-mediated carbon monoxide deoxygenation and coupling: mono- and dicarbyne complexes precede C–O bond cleavage and C–C bond formation. J. Am. Chem. Soc..

[14] Sazama GT, Betley TA (2011). Reductive coupling of CO templated by iron bound to the tris(pyrrolide)ethane scaffold. Organometallics.

[15] Coffin VL, Brennen W, Wayland BB (1988). Thermodynamic studies of competitive adduct formation: single- and double-insertion reactions of carbon monoxide with rhodium octaethylporphyrin dimer. J. Am. Chem. Soc..

[16] Bianconi PA, Williams ID, Engeler MP, Lippard SJ (1986). Reductive coupling of two carbon monoxide ligands to form a coordinated alkyne. J. Am. Chem. Soc..

[17] Miller AJM, Labinger JA, Bercaw JE (2010). Homogeneous CO hydrogenation: ligand effects on the lewis acid-assisted reductive coupling of carbon monoxide. Organometallics.

[18] Miller AJM, Labinger JA, Bercaw JE (2010). Homogeneous CO hydrogenation: dihydrogen activation involves a frustrated lewis pair instead of a platinum complex. J. Am. Chem. Soc..

[19] Miller AJM, Labinger JA, Bercaw JE (2008). Reductive coupling of carbon monoxide in a rhenium carbonyl complex with pendant lewis acids. J. Am. Chem. Soc..

[20] Wayland, B. B., Sherry, A. E. & Coffin, V. L. Double-stranded RNA drives SARS-CoV-2 nucleocapsid protein to undergo phase separation at specific temperatures. *J. Chem. Soc., Chem. Commun.* 662–663 (1989). 10.1093/nar/gkac596 PMC9371935 35871289

[21] Chatani N, Shinohara M, Ikeda S-I, Murai S (1997). Reductive oligomerization of carbon monoxide by rhodium-catalyzed reaction with hydrosilanes. J. Am. Chem. Soc.

[22] Tsoureas N, Summerscales OT, Cloke FGN, Roe SM (2013). Steric effects in the reductive coupling of CO by mixed-sandwich uranium(III) complexes. Organometallics.

[23] Arnold PL, Turner ZR, Bellabarba RM, Tooze RP (2011). Carbon monoxide coupling and functionalisation at a simple uranium coordination complex. Chem. Sci.

[24] Shima T, Hou Z (2006). Hydrogenation of carbon monoxide by tetranuclear rare earth metal polyhydrido complexes. selective formation of ethylene and isolation of well-defined polyoxo rare earth metal clusters. J. Am. Chem. Soc.

[25] Des Abbayes, H. & Salaun, J. Y. Double carbonylation and beyond: systems at work and their organometallic models. *Dalton Trans*. 1041–1052 10.1039/B209103H (2003).

[26] Trzupek LS, Newirth TL, Kelly EG, Sbarbati NE, Whitesides GM (1973). Mechanism of reaction of carbon monoxide with phenyllithium. J. Am. Chem. Soc.

[27] Nudelman, N. & Schulz, H. Synthesis of highly hindered 1,2-diaryl diketones and of cis- and trans-1,2-diacetoxy-1,2-bis(aryl)ethenes. *J. Chem. Res*. (S), 422–423 (1999).

[28] Nudelman NS, Outumuro P (1982). Insertion of carbon monoxide into carbon-lithium bonds. a convenient one-step synthesis of 1,2-diketone diaryl derivatives. J. Organomet. Chem..

[29] Goldberg KI, Bergman RG (1989). Synthesis of dialkyl- and alkyl(acyl)rhenium complexes by alkylation of anionic rhenium complexes at the metal center. mechanism of a double carbonylation reaction that proceeds via the formation of free methyl radicals in solution. J. Am. Chem. Soc..

[30] Amadio E, Freixa Z, van Leeuwen PWNM, Toniolo L (2015). Palladium catalyzed oxidative carbonylation of alcohols: effects of diphosphine ligands. Catal. Sci. Technol..

[31] Amadio E, Cavinato G, Dolmella A, Toniolo L (2010). Catalytic properties of [Pd(COOMe)_*n*_X_2−*n*_(PPh_3_)_2_] (*n* = 0, 1, 2; X = Cl, NO_2_, ONO_2_, OAc and OTs) in the oxidative carbonylation of MeOH. Inorg. Chem.

[32] Pawlow JH, Sadow AD, Sen A (1997). Palladium(II)-catalyzed carbonylation of alkane dinitrite esters to polyoxalates. Organometallics.

[33] Current SP (1983). Catalytic synthesis of oxalate esters. J. Organomet. Chem.

[34] Rivetti F, Romano U (1979). Alcohol carbonylation with palladium(II) complexes, effects of ligands, carbon monoxide, pressure and added bases. J. Organomet. Chem.

[35] Fenton DM, Steinwand PJ (1974). Noble metal catalysis. III. preparation of dialkyl oxalates by oxidative carbonylation. J. Organomet. Chem.

[36] Hansgen D, Vlachos D, Chen J (2010). Using first principles to predict bimetallic catalysts for the ammonia decomposition reaction. Nat. Chem..

[37] Schuler E, Demetriou M, Shiju NR, Gruter G-JM (2021). Towards sustainable oxalic acid from CO_2_ and biomass. ChemSusChem.

[38] Swamy PC, Solel E, Reany O, Keinan E (2018). Synthetic evolution of the multifarene cavity from planar predecessors. Chem. Eur. J..

[39] Xu R, Chakraborty S, Yuan H, Jones WD (2015). Acceptorless, reversible dehydrogenation and hydrogenation of N-heterocycles with a cobalt pincer catalyst. ACS Catal.

[40] Yoo C, Kim J, Lee Y (2013). Synthesis and reactivity of nickel(II) hydroxycarbonyl species, NiCOOH-κC. Organometallics.

[41] Lee, D. W., Jensen, C. M. & Morales-Morales, D. Reactivity of iridium PCP pincer complexes toward CO and CO_2_ crystal structures of IrH(κ_2_-O_2_COH){C_6_H_3_−2,6-(CH_2_PBut_2_)_2_} and IrH(C(O)OH){C_6_H_3_−2,6-(CH_2_PBut_2_)_2_}·H_2_O). *Organometallics* **22**, 4744–4749 (2003).

[42] Luo J, Rath NP, Mirica LM (2011). Dinuclear Co(II)Co(III) mixed-valence and Co(III)Co(III) complexes with N- and O-donor ligands: characterization and water oxidation studies. Inorg. Chem.

[43] Zhou XT, Day AI, Edwards AJ, Willis AC, Jackson WG (2005). Facile C-H bond activation: synthesis of the N_4_C donor set pentadentate ligand 1,4-bis(2-pyridylmethyl)−1,4-diazacyclononane (dmpdacn) and a structural study of its alkyl-cobalt(III) complex Co(dmpdacn-*C*)(OH_2_) (ClO_4_)_2_·H_2_O and its hydroxylated derivative Co(dmpdacnOH-*O*)Cl(ClO_4_)_2_·C_3_H_6_O. Inorg. Chem.

[44] Hikichi S, Yoshizawa M, Sasakura Y, Akita M, Morooka Y (1998). First synthesis and structural characterization of dinuclear M(III) bis(*μ*-oxo) complexes of nickel and cobalt with hydrotris(pyrazolyl)borate ligand. J. Am. Chem. Soc.

[45] Cook BJ, Di Francesco GN, Abboud KA, Murray LJ (2018). Countercations and solvent influence CO_2_ reduction to oxalate by chalcogen-bridged tricopper cyclophanates. J. Am. Chem. Soc.

[46] Clark RJH, Firth S (2002). Raman, infrared and force field studies of K_2_^12^C_2_O_4_·H_2_O and K_2_^13^C_2_O_4_·H_2_O in the solid state and in aqueous solution, and of (NH_4_)_2_^12^C_2_O_4_·H_2_O and (NH_4_)_2_^13^C_2_O_4_·H_2_O in the solid state. spectrochim. Acta, Part A.

[47] Neese F (2012). The ORCA program system. Wiley Interdiscip. Rev.: Comput. Mol. Sci.

[48] Neese, F. Software up-date: The ORCA program system version 5.0. *Wiley Interdiscip. Rev.: Comput. Mol. Sci*. e1606 (2022).

[49] Brandenburg, J. G., Bannwarth, C., Hansen, A. & Grimme, S. *J. Chem. Phys* **148**, 064104 (2018). 10.1063/1.5012601 29448802

[50] Chan B, Dawson W, Nakajima T (2022). Searching for a reliable density functional for molecule-environment interactions, found B97M-V/def2-mTZVP. J. Phys. Chem. A.

