# Supplementary material for: Direct synthesis of oxalic acid via oxidative CO coupling mediated by a dinuclear hydroxycarbonylcobalt(III) complex
Source: Nat Commun. 2023 May 12;14:2739. doi: 10.1038/s41467-023-38442-4 (PMC10182058; doi:10.1038/s41467-023-38442-4)
Supplement: Supplementary file 1 — Supplementary Information [file 41467_2023_38442_MOESM1_ESM.pdf]

Supplementary Information for

Direct synthesis of oxalic acid via oxidative CO coupling mediated by a dinuclear hydroxycarbonylcobalt(III) complex

Yingzhuang Xu[1], Songyi Li[1], Huayi Fang[1]*

Affiliation:

[1]School of Materials Science and Engineering, Tianjin Key Lab for Rare Earth Materials and Applications, Nankai University, Tianjin 300350, China.

*Corresponding author. Email: hfang@nankai.edu.cn.

Table of Contents

General information. S4

General procedure for the catalytic production of oxalic acid from CO S4

General procedure for product detection S5

Assignment of the spin state of Co(II)/Co(III) centers in complexes 1 and 2 S5

Computational details. S6

Supplementary Fig. 1. ^{1}H NMR spectrum of the reaction solution for the synthesis of **1** in *d*6-DMSO. S7

Supplementary Fig. 2. Plot of χ_MT vs T for **1**. S8

Supplementary Fig. 3. *XPS* measurement of **1**. S9

Supplementary Fig. 4. Unrestricted corresponding orbitals (with overlap coefficient equals 0) of **1** (a) and **2** (b), and spin density plots of **1** (c) and **2** (d). S10

Supplementary Fig. 5. *XPS* measurement of **2**. S11

Supplementary Fig. 6. Plot of χ_MT vs T for **2**. S12

Supplementary Fig. 7. *XPS* measurement of **3**. S13

Supplementary Fig. 8. ^{1}H NMR spectrum of **H_2L** in $CDCl_3$. S14

Supplementary Fig. 9. ^{13}C NMR spectrum of **H_2L** in $CDCl_3$. S15

Supplementary Fig. 10. ^{1}H NMR spectrum of **3** in CD_3OD. S16

Supplementary Fig. 11. ^{13}C NMR spectrum of **3** in CD_3OD. S17

Supplementary Fig. 12. *XPS* measurement of **4**. S18

Supplementary Fig. 13. IR spectra of **4** prepared using ^{12}CO (red) and ^{13}CO (blue). S19

Supplementary Fig. 14. ^{1}H NMR spectrum of **4** in DMSO. S20

Supplementary Fig. 15. ^{13}C NMR spectrum of **4** in *d*6-DMSO. S21

Supplementary Fig. 16. ^{13}C NMR spectrum of ^{13}C-labelled **4** in *d*6-DMSO. S22

Supplementary Fig. 17. TGA curve of **4** in nitrogen atmosphere. S23

Supplementary Fig. 18. *XPS* measurement of **5**. S24

Supplementary Fig. 19. Plot of χ_MT vs T for **5**. S25

Supplementary Fig. 20. Unrestricted corresponding orbitals (with overlap coefficient equals 0) of **5** (a) and calculated spin density of **5** (b). S26

Supplementary Fig. 21. The plausible generation pathway of **5** from the proposed dinuclear hydroxylcobalt(III) intermediate. S27

Supplementary Fig. 22. ^{1}H NMR spectrum of precipitates formed during the production of oxalic acid catalyzed by **4** in *d*6-DMSO. S28

Supplementary Fig. 23. *XPS* measurement of the precipitates formed during the production of oxalic acid catalyzed by **4**. S29

Supplementary Fig. 24. IR spectra of $Ca^{13}C_2O_4$ (a, obtained by adding $CaCl_2$ to the reaction solution for the catalytic production of oxalic acid from ^{13}CO) and $Ca^{12}C_2O_4$ (b). S30

Supplementary Fig. 25. The MS measurements (negative mode) of $H_2C_2O_4$ standard reagent (a), and $H_2C_2O_4$ produced using $H_2{}^{18}O/{}^{16}O_2$ (b) and $H_2{}^{16}O/{}^{18}O_2$ (c). S31

Supplementary Fig. 26. UV-Vis spectra of **1-5** in ethanol. S32
Supplementary Fig. 27. Detection of H_2O_2 generated in the production of oxalic acid catalyzed by **3** (red), **4** (blue) and **5** (magenta) using iodometry method. S33
Supplementary Fig. 28. H_2O_2 detection based on neocuproine/$CuSO_4$ titration: (a) UV-Vis spectra recorded for the titration of H_2O_2 standard samples; (b) UV-Vis spectra recorded for the titrations of H_2O_2 formed in the production of oxalic acid catalyzed by **3** (red), **4** (blue) and **5** (olive). S34
Supplementary Fig. 29. H_2O_2 detection based on Cerium sulfate titration: (a) UV-Vis spectra recorded for the titration of H_2O_2 standard samples; (b) UV-Vis spectra recorded for the titrations of H_2O_2 formed in the production of oxalic acid catalyzed by **3** (red), **4** (blue) and **5** (olive). S35
Supplementary Fig. 30. Plausible light-promoted pathways for the formation of **4** from dinuclear hydroxocobalt(III) complex. S36
Supplementary Fig. 31. Gibbs free energy profiles for the generations of oxalic acid from **4** via the hydroxycarbonyl radical attack based pathway (bond lengths and distances are provided in Å). S37
Supplementary Fig. 32. Spin density plots of **IN 5-16** and **TS 5-8**. S38
Supplementary Fig. 33. The structures of the minimal energy crossing points (MECPs) of **IN 11** / **IN 12** (a) and **IN 14** / **IN 15** (b). S39
Supplementary Table 1. Summary of crystallographic data collection and structure refinement for **1-5**. S41
Supplementary Fig. 34. ORTEP representations (50% probability) of **1.** S42
Supplementary Fig. 35. ORTEP representations (50% probability) of **2**. S43
Supplementary Fig. 36. ORTEP representations (50% probability) of **3**. S44
Supplementary Fig. 37. ORTEP representations (50% probability) of **4**. S45
Supplementary Fig. 38. ORTEP representations (50% probability) of **5**. S46
Supplementary Table 2. Selected bond distances (Å) and angles (deg) of the solid state structures of **1-5**. S47
Supplementary Table 3. Comparisons of the RMSDs of the selected bonds (labelled in blue) and calculated C=O stretching frequencies of **4** using different density functionals. S49
References. S50

General information

Materials. All manipulations involving air-sensitive materials were performed under N_2 atmosphere using standard Schlenk techniques or in gloveboxes. Chemicals were purchased from Sigma-Aldrich, Alfa Aesar, or J&K Scientific Ltd. and used without further treatment unless otherwise noted. Deuterated solvents (CD_3OD, DMSO and $CDCl_3$) were purchased from Cambridge Isotope Laboratory Inc, and used without further treatment. The macrocyclic ligand (H_2L) was synthesized by literature procedures[1].

Physical Measurements. ^1H NMR and ^{13}C NMR spectra were recorded on a Bruker Ascend^{TM} 400 spectrometer at 298 K, and the chemical shifts were referenced to solvent residual signals. The common glass NMR tubes with an outside diameter of 5 mm (Wilmad WG-1000) were used for diamagnetic compounds, and J Young tubes (Wilmad GVA-5, with an outside diameter of 5 mm) were used for samples for which specific atmosphere were required. For ^1H NMR measurements, 0.0020 ~ 0.0030 mg of the sample was dissolved in 0.4 mL of deuterated reagents in the NMR tube and then the measurement was conducted; For ^{13}C NMR measurements, 0.0050 ~ 0.0100 mg of the sample was dissolved in 0.4 mL of deuterated reagents in the NMR tube and then the measurement was conducted.

The UV-vis measurements were conducted on a Shimadzu 2600 spectrometer. The infrared spectra were recorded using a Bruker Tensor 37 spectrometer. The XPS spectra were recorded on an ESCALAB 250 instrument. The X-band CW-EPR measurements were recorded on a Bruker EMX plus spectrometer at 97 K. Thermal analysis was carried out on Netzsch TG209 thermobalance under a N_2 atmosphere. Magnetization measurements were carried out using a Quantum Design MPMS 3 SQUID magnetometer equipped with a 5T magnet. The elemental analysis results were obtained by a Vario EL cube analyzer. The GC measurements of liquid samples were performed on Shimadzu GC-2010 Pro instrument equipped with a flame ionization detector and a WondaCap Wax capillary column (30 m × 0.25 mm). Gas samples for GC measurements were analyzed using a Shimadzu 2014C equipped with a thermal conductivity detector and a custom column (Porapak-N 3.0 m×3.2 mm×2.1 mm). The formic acid product was determined and quantified by using a 400 MHz liquid NMR spectrometer. The amount of oxalic acid product was detected by LCMS, performed on Shimadzu LCMS-2020 instrument equipped with a UV detector using a Shim-Pack Scepter C18-120 reverse column (3 μm, 3×33 mm). The mobile phases were acetonitrile and water/5 mM NH_4HCO_3 solution, and the flow ratio was 5:95 at a total flow rate of 1.5 mL^{-1} min. In addition, oxalic acid was quantified using the time-honored method of precipitation by $CaCl_2$. Light in the irradiation experiments were provided by a 500 W high voltage xenon lamp (CEL-HXF300-T3, Beijing China Education Au-light Co.,Ltd.,).

General procedure for the catalytic production of oxalic acid from CO

4.0 mL of methanol solution containing 0.0170 mmol of catalysts (complexes **1-5**) was transferred into a 25.0 mL Schlenk flask. After three freeze-pump-thaw cycles,

1 atm of O_2 was inflated into the Schlenk flask, followed by the addition of 1 atm of CO. The Schlenk flask was set 20.0 cm aside a 500 W xenon lamp at 30 °C for 28 h.

^{13}C-labelling experiment for the catalytic production of oxalic acid. 0.0170 mmol of complex **4-^{13}C** was added into a 25.0 mL Schlenk flask with 4.0 mL methanol. After three freeze-pump-thaw cycles, 1 atm of O_2 was inflated into the Schlenk flask, followed by the addition of 1 atm of ^{13}CO. The Schlenk flask was set 20.0 cm aside a 500 W xenon lamp at 30 °C. After stirring the reaction mixture for 28 h, 0.680 mmol of $CaCl_2$ was added to the reaction solution, and the resulted white precipitate was collected and used for IR analysis.

General procedure for product detection

Formic acid detection: 4.0 mL of methanol solution containing 0.0170 mmol of complex **4** was transferred into a 25.0 mL Schlenk flask. After three freeze-pump-thaw cycles, 1 atm of O_2 was inflated into the Schlenk flask, followed by the addition of 1 atm of CO. The Schlenk flask was set 20.0 cm aside a 500 W xenon lamp at 30 °C for 28 h. Then, excess amount of NaOH was added to the reaction solution at room temperature in an air atmosphere, and then the reaction solution was dried and used for NMR analysis.

Oxalic acid detection: 4.0 mL of methanol solution containing 0.0170 mmol of complex **4** was transferred into a 25.0 mL Schlenk flask. After three freeze-pump-thaw cycles, 1 atm of O_2 was inflated into the Schlenk flask, followed by the addition of 1 atm of CO. The Schlenk flask was set 20.0 cm aside a 500 W xenon lamp at 30 °C for 28 h. The precipitate was filtered off and the filtrate was directly analyzed by LCMS at room temperature in an air atmosphere. (The amount of oxalic acid product was detected by LCMS, performed on Shimadzu LCMS-2020 instrument equipped with a UV detector using a Shim-Pack Scepter C18-120 reverse column (3 μm, 3×33 mm). The mobile phases were acetonitrile and water/5 mM NH_4HCO_3 solution, and the flow ratio was 5:95 at a total flow rate of 1.5 mL/min.)

CO_2 detection: 4.0 mL of methanol solution containing 0.0170 mmol of complex **4** was transferred into a 25.0 mL Schlenk flask. After three freeze-pump-thaw cycles, 1 atm of O_2 was inflated into the Schlenk flask, followed by the addition of 1 atm of CO. The Schlenk flask was set 20.0 cm aside a 500 W xenon lamp at 30 °C for 28 h. After the reaction, the gas is collected by balloon and then connected to GC injector for gas analysis.

Dimethyl oxalate, dimethyl carbonate and methyl formate detection: 4.0 mL of methanol solution containing 0.0170 mmol of complex **4** was transferred into a 25.0 mL Schlenk flask. After three freeze-pump-thaw cycles, 1 atm of O_2 was inflated into the Schlenk flask, followed by the addition of 1 atm of CO. The Schlenk flask was set 20.0 cm aside a 500 W xenon lamp at 30 °C for 28 h. The precipitate was filtered off and the filtrate was then analyzed by GC.

Assignment of the spin state of Co(II)/Co(III) centers in complexes 1-2

The calculated spin density for **1** showed that 2.58 unpaired electrons were found

on each of the cobalt centers, consistent with the high spin (S = 3/2) state for both of the Co(II) centers. Unrestricted corresponding orbital analysis of **1** is also in line with the assignment of two high spin Co(II) centers. The spin density analysis of **2**, as shown in Supplementary Fig. 4, showed that 2.61 unpaired electrons were located on the Co(II) center. Meanwhile, unrestricted corresponding orbital analysis of **2** confirms the presence of a total of three unpaired electrons on the Co(II) center. Given all the aforementioned results, the assignment of a high spin (S = 3/2) Co(II) center and a low spin (S = 0) Co(III) center was made.

Computational details

All calculations were performed on the ORCA quantum chemistry program package (version 5.0.3). The B97-3c calculation setup developed by Grimme and coworkers was applied[2]. This setup is based on the B97 GGA functional and includes D3 with three-body contribution and a short-range bond length correction. The modified, stripped-down triple-ζ basis, def2-mTZVP[3] is used in the setup. The solvation effect was considered using the conductor-like polarizable continuum model (C-PCM) [4]. Structure optimizations were performed without any geometrical constraint. For the relatively large complex **5** with a total of 174 atoms, which is nearly twice the size of other dicobalt complexes, the crystal structure with the optimized positions for hydrogen atoms was used for the unrestricted corresponding orbital and spin density analysis.

The carbon-nitrogen bond lengths are used as the indexes to distinguish imine moietes (C=N bonds) from amine moieties (C-N bonds). Both of the imine and amine moieties are found in H_2L ligand (CCDC 2235093), with bond lengths of 1.272-1.280 Å and 1.445–1.447 Å, respectively. For the dicobalt complexes **1-5**, all the carbon-nitrogen bond lengths are in the range of 1.280-1.293 Å, which are very comparable to the bond lengths of the imine groups in H_2L ligand and typical values (1.280-1.284 Å) reported for other imine compounds[5] . In addition, the resonances of protons in both the amine (-C$\boldsymbol{H_2}$-NH-) and imine (-C***H***=N-) moieties were seen in the ^{1}H NMR spectrum of H_2L ($\delta \sim 4.45$ ppm for the 4 protons in amine moieties and $\delta \sim 8.63$ ppm for the 2 protons in imine moieties, Supplementary Fig. 8). No -C$\boldsymbol{H_2}$-NH- signal was observed in ^{1}H NMR spectra of the obtained diamagnetic dicobalt complexes (Supplementary Fig. 10 and Supplementary Fig. 14). These results indicated that the amine moieties in the free base H_2L ligand were converted to imine moieties during the metallation.

Without the addition of $Co(OAc)_2$, no conversion or decomposition of the H_2L ligand was observed under identical conditiaons to the synthesis of dicobalt(II) complex **1**. To confirm the generation of H_2 during the synthesis of complex **1**, the reaction was performed in a sealed J. Young NMR tube in *d*$_6$-DMSO at 80 °C. The recorded ^{1}H NMR spectrum of the reaction solution showed a singlet resonance at $\delta = 4.35$ ppm that is characteristic for H_2 (Supplementary Fig. 1). Similiar cobalt mediated dehydrogenation of amines was also known[6].

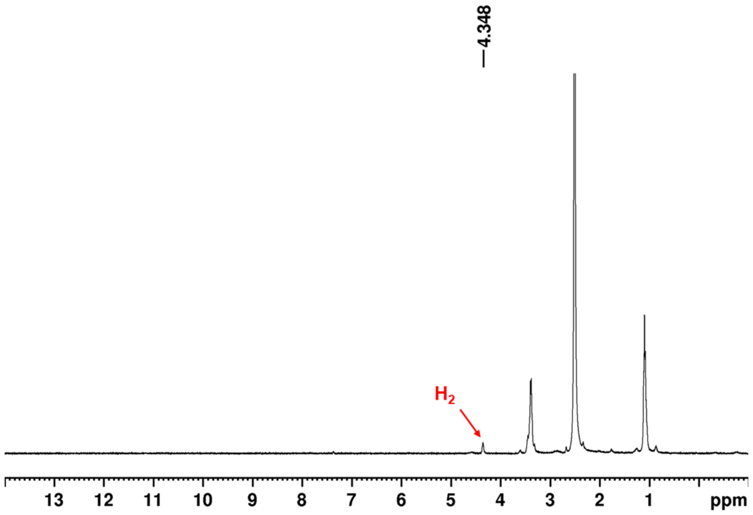

Supplementary Fig. 1. ^{1}H NMR spectrum of the reaction solution for the synthesis of **1** in *d*$_6$-DMSO.

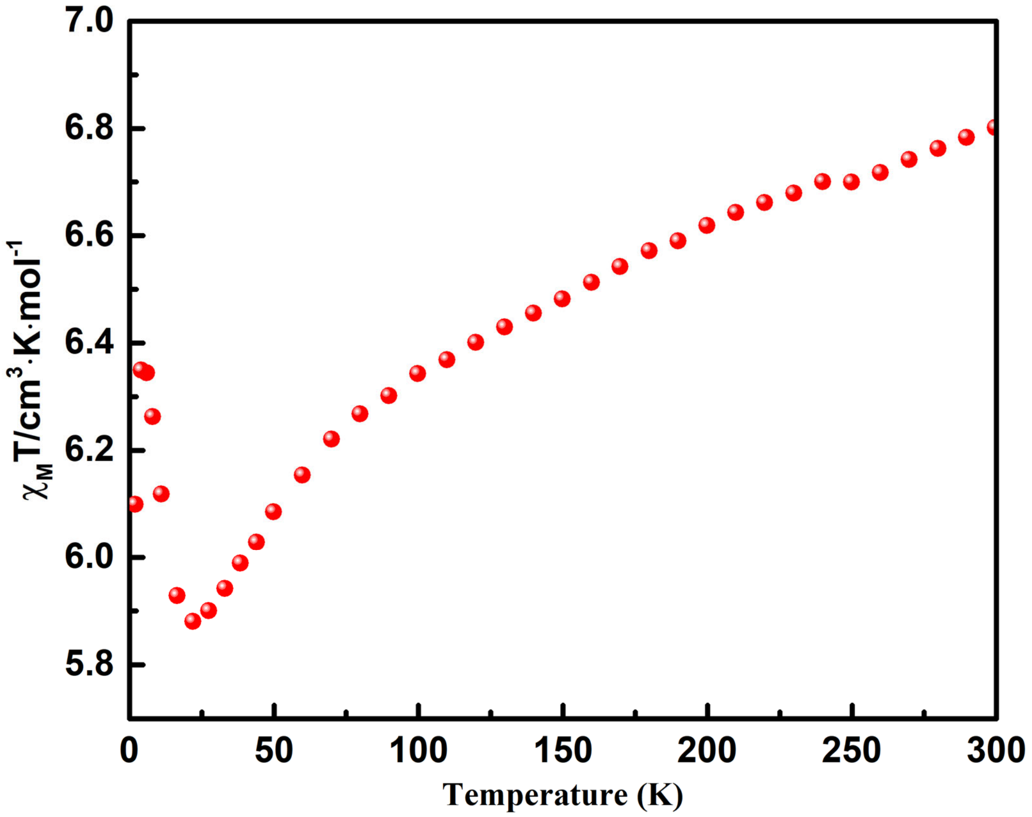

Supplementary Fig. 2. Plot of χ_MT vs T for **1**.

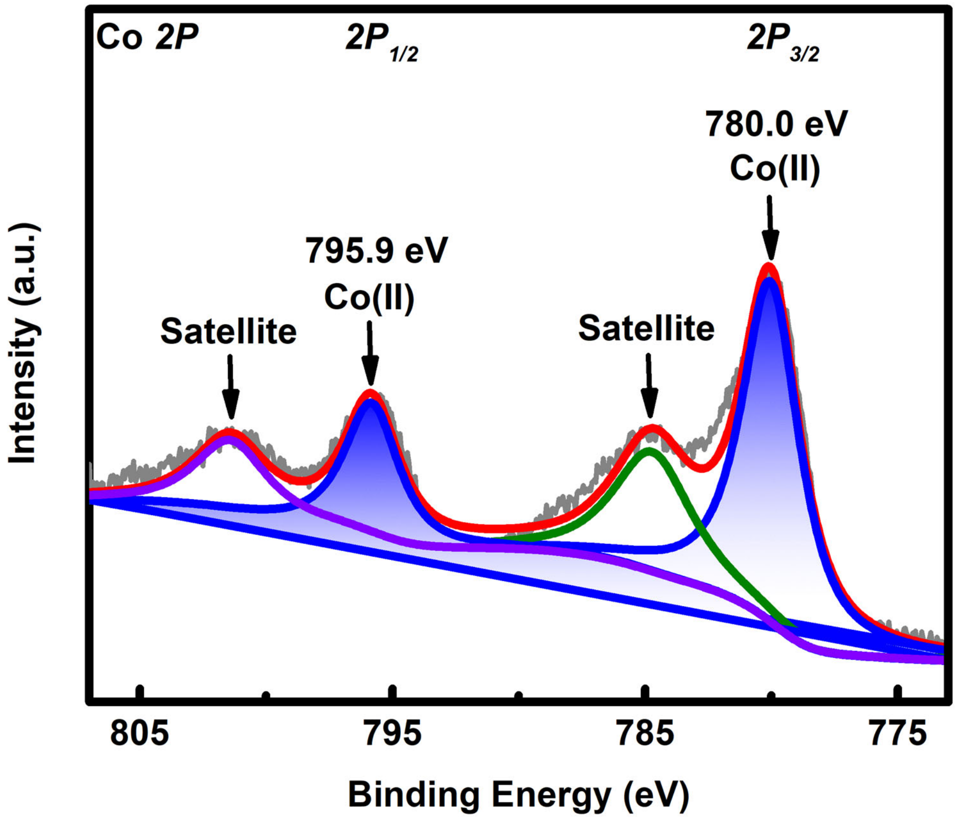

Supplementary Fig. 3. *XPS* measurement of **1**.

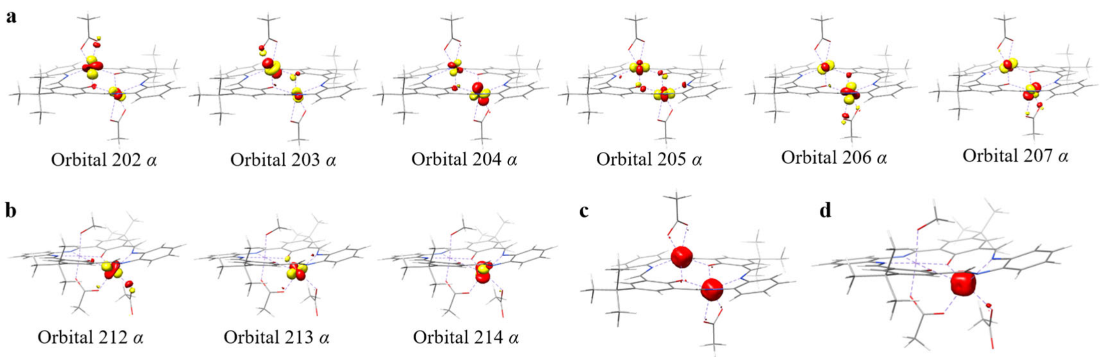

Supplementary Fig. 4. Unrestricted corresponding orbitals (with overlap coefficient equals 0) of **1** (a) and **2** (b), and spin density plots of **1** (c) and **2** (d).

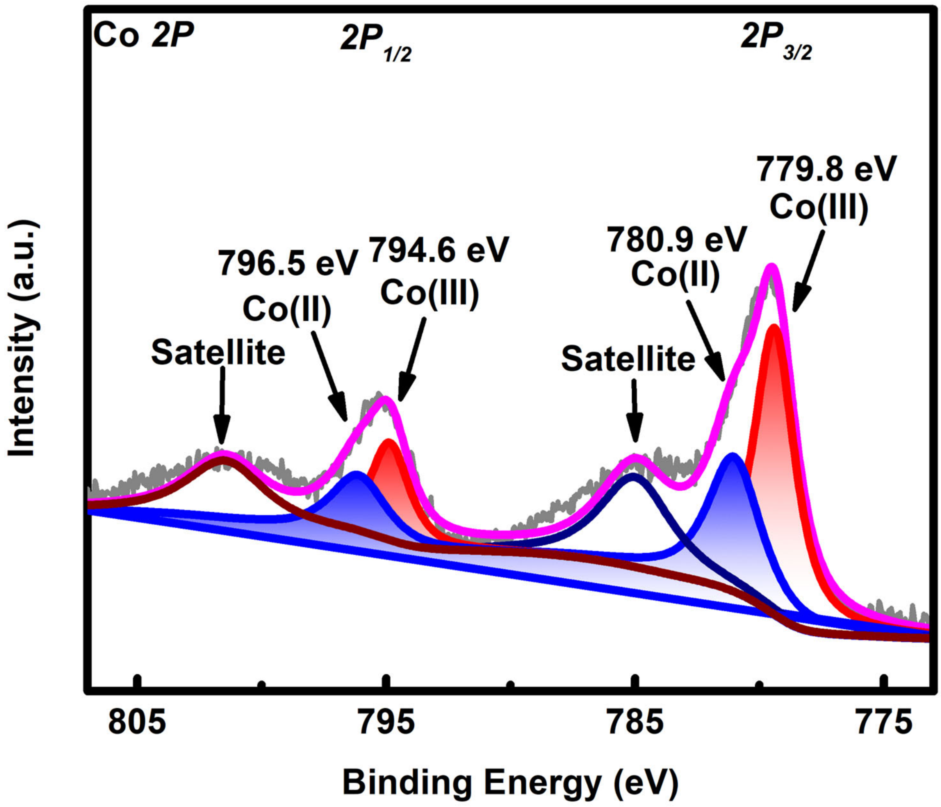

Supplementary Fig. 5. *XPS* measurement of **2**.

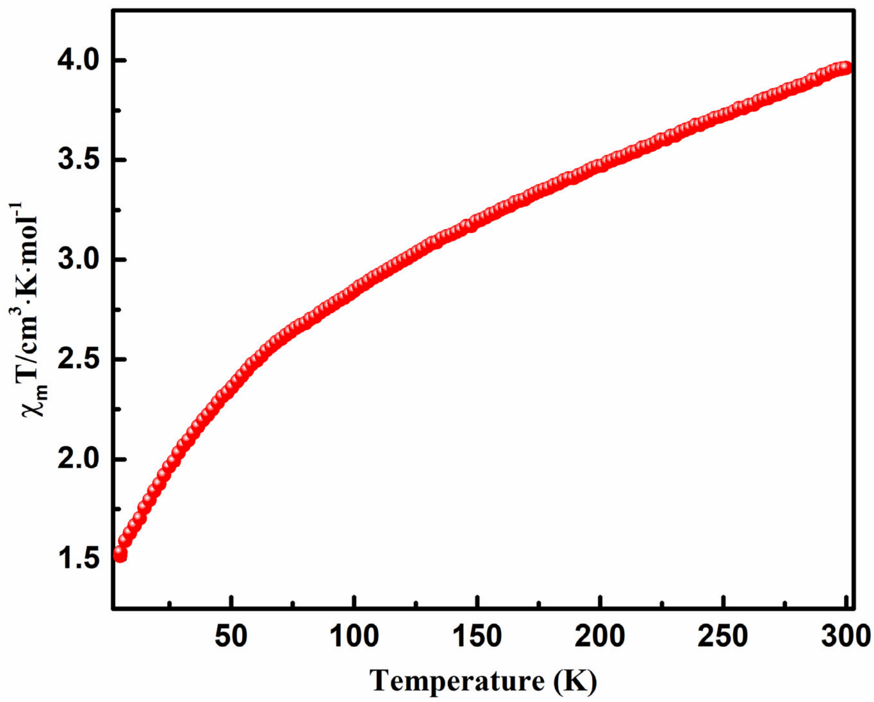

Supplementary Fig. 6. Plot of χ_MT vs T for **2**.

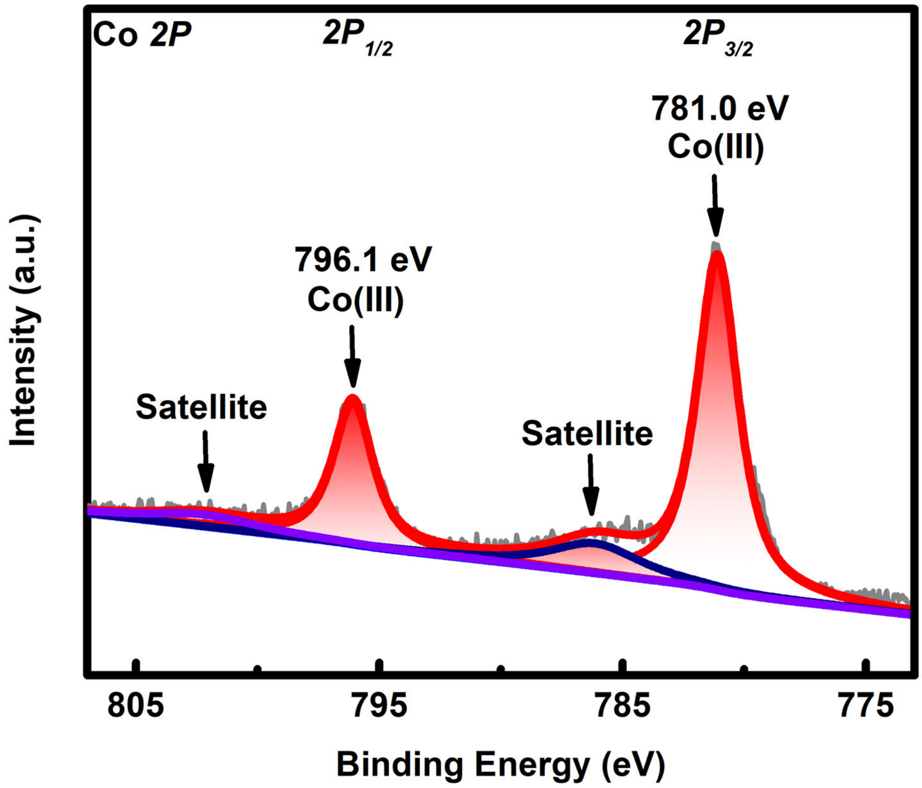

Supplementary Fig. 7. *XPS* measurement of **3**.

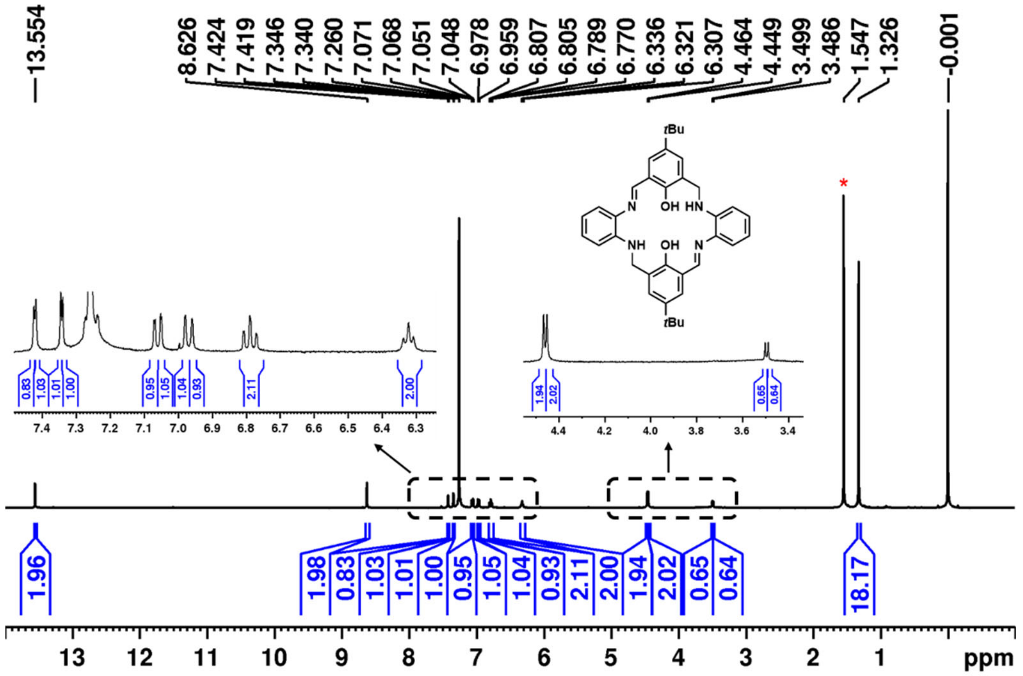

Supplementary Fig. 8. ^{1}H NMR spectrum of **H_2L** in $CDCl_3$. (* denotes the resonance of H_2O)

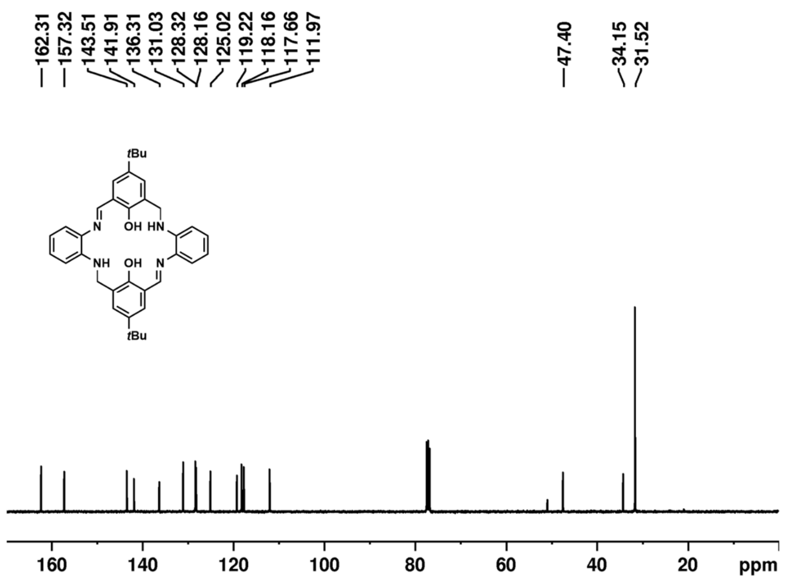

Supplementary Fig. 9. ^{13}C NMR spectrum of **H_2L** in $CDCl_3$.

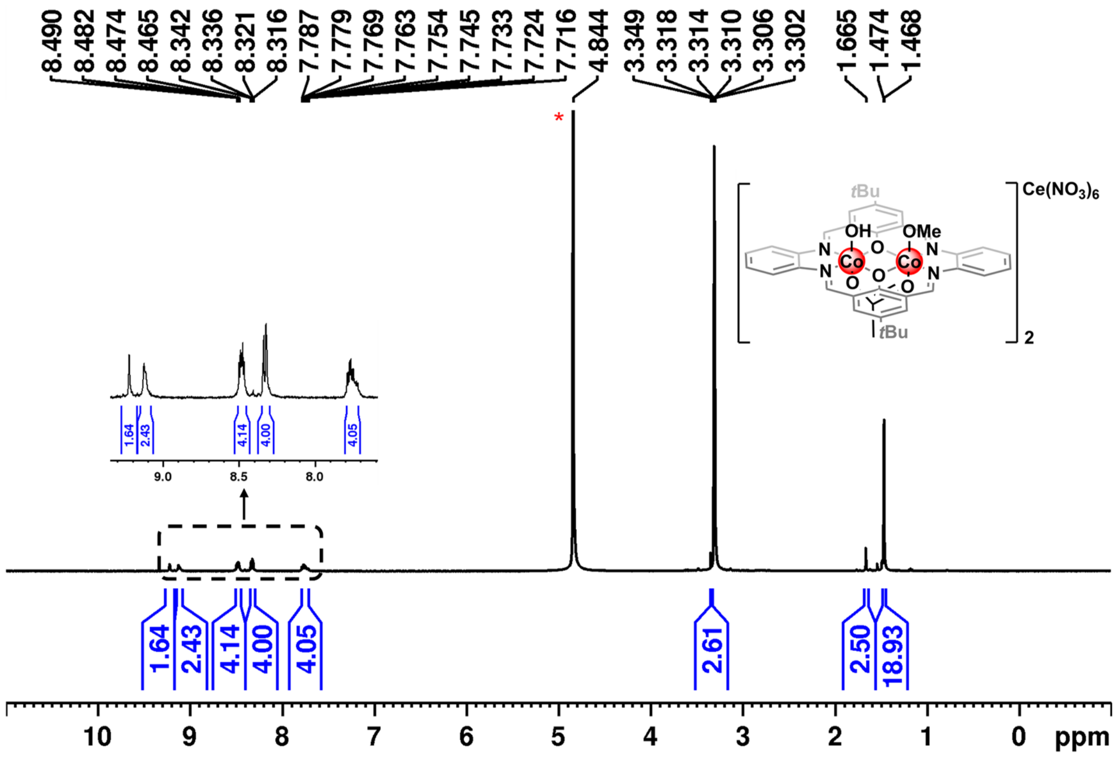

Supplementary Fig. 10. ^{1}H NMR spectrum of **3** in CD_3OD. (* denotes the resonance of H_2O)

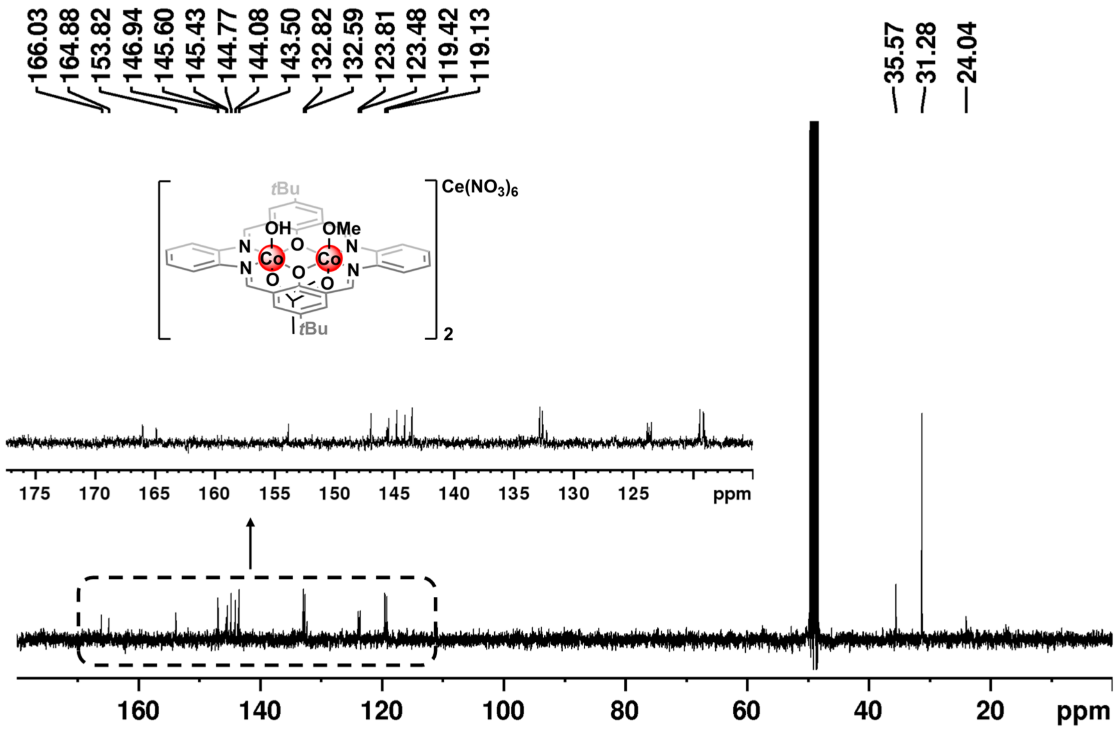

Supplementary Fig. 11. ^{13}C NMR spectrum of **3** in CD_3OD.

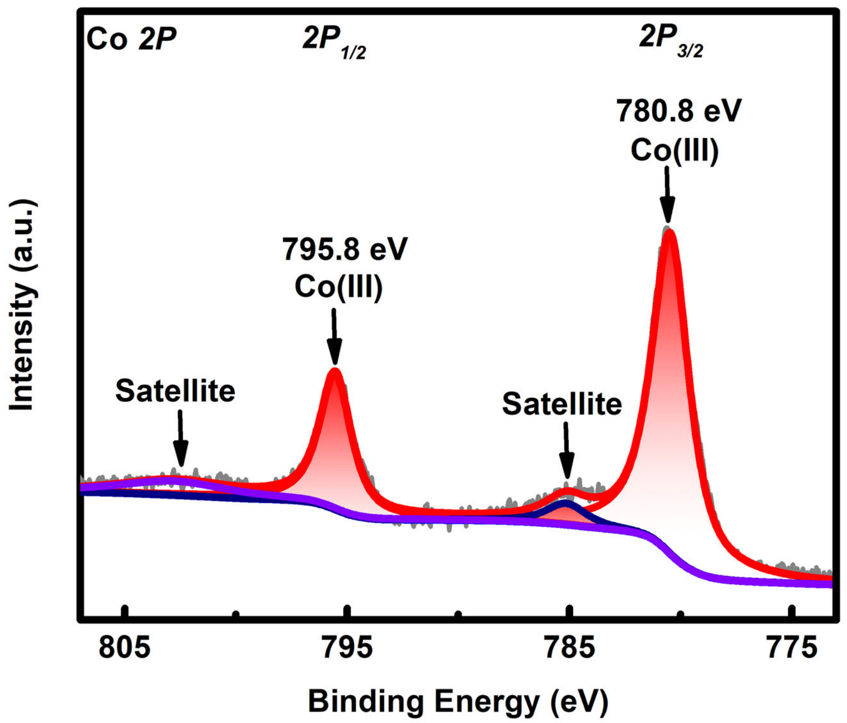

Supplementary Fig. 12. *XPS* measurement of **4**.

Procedure of the ^{13}C-labelling experiment: In a 25.0 mL Schlenk flask, a solution of complex **3** (0.0350 g, 0.0170 mmol) in 4.0 mL methanol was degassed by three freeze-pump-thaw cycles. 1 atm of O_2 was inflated into the Schlenk flask, followed by the addition of 1 atm of ^{13}CO. The solution was stirred for 24 h at 50 °C. The red precipitate was collected by filtration and sent for the IR analysis.

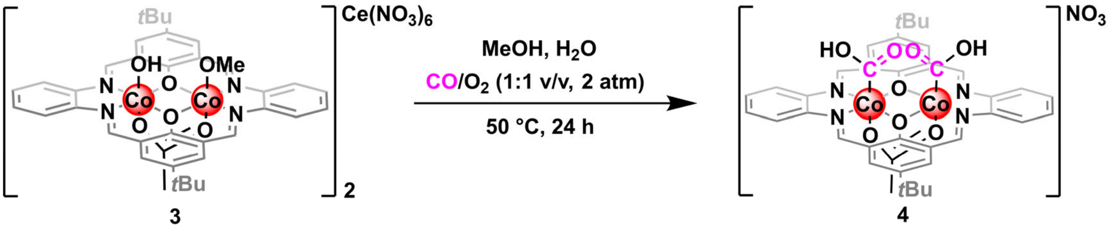

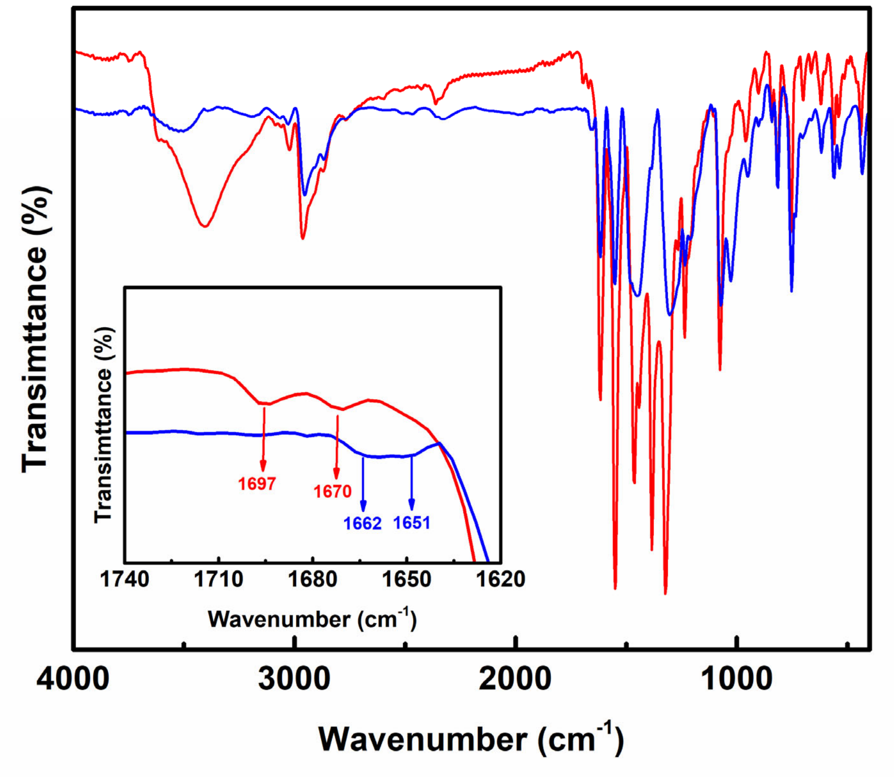

Fig. 13. IR spectra of **4** prepared using ^{12}CO (red) and ^{13}CO (blue).

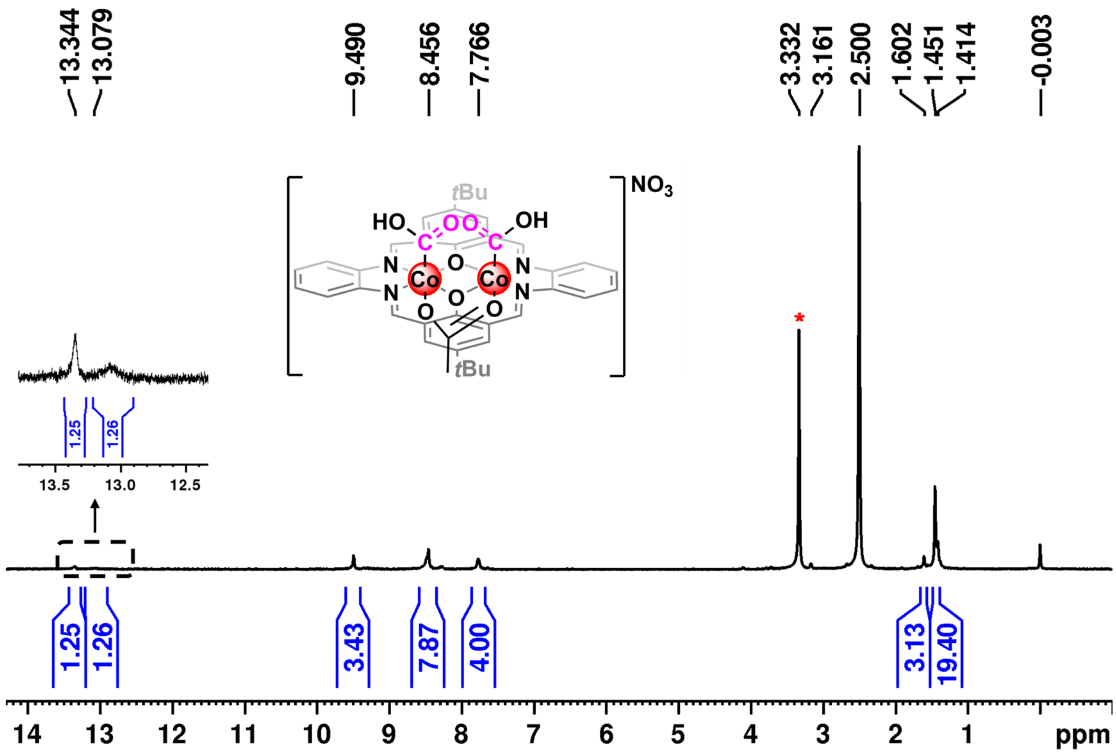

Supplementary Fig. 14. ^{1}H NMR spectrum of **4** in *d*$_6$-DMSO. (* denotes the resonance of H_2O)

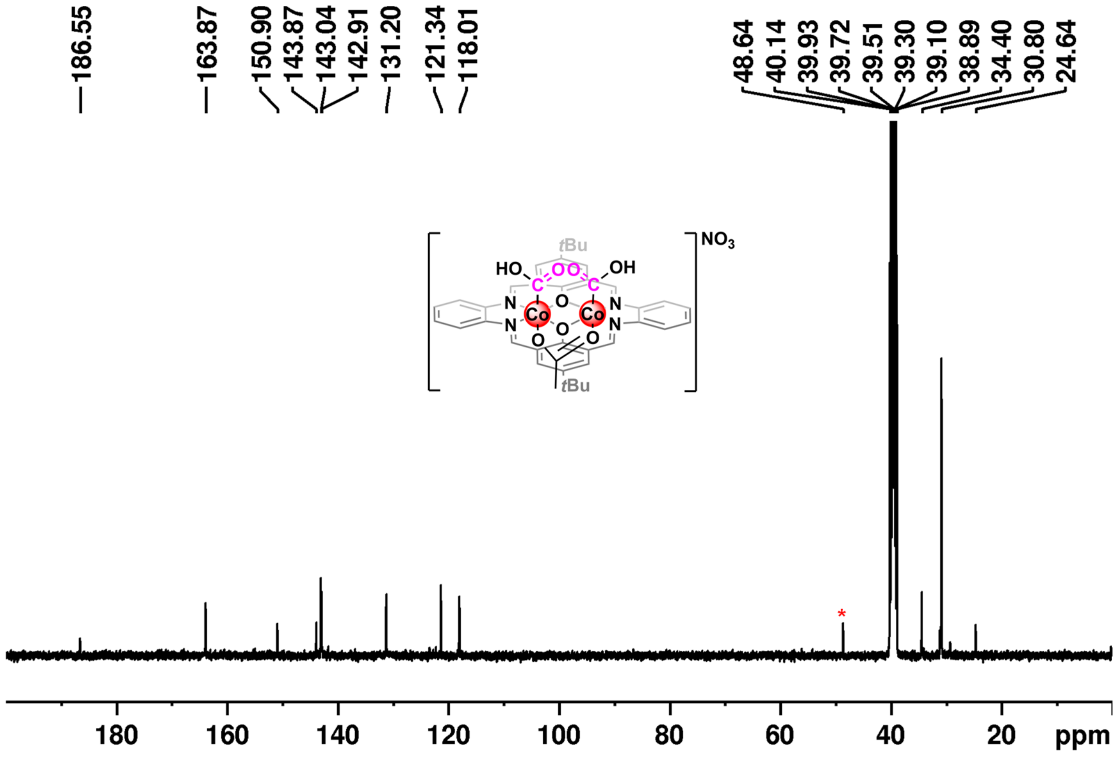

Supplementary Fig. 15. ^{13}C NMR spectrum of **4** in *d*$_6$-DMSO. (* denotes the resonance of MeOH)

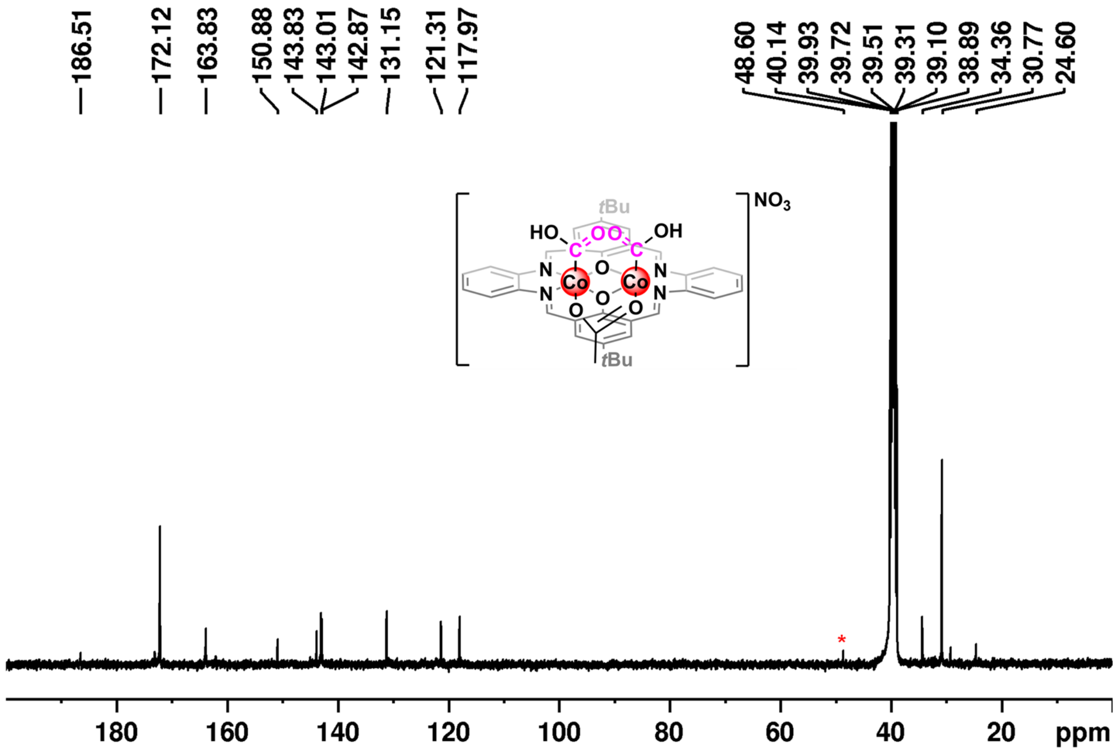

Supplementary Fig. 16. ^{13}C NMR spectrum of ^{13}C-labelled **4** in *d₆*-DMSO. (* denotes the resonance of MeOH)

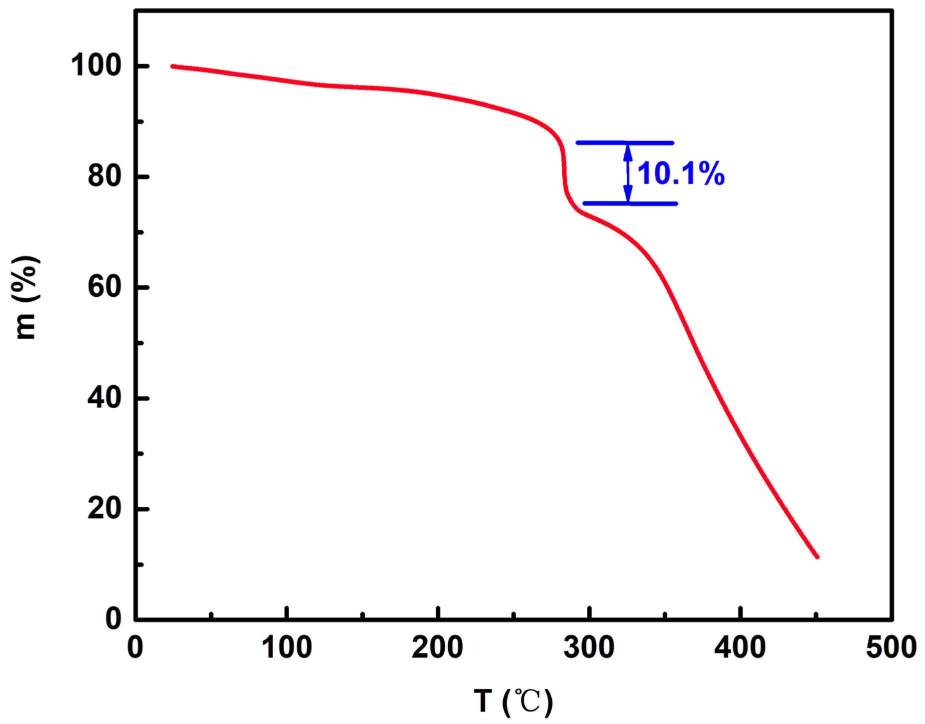

Supplementary Fig. 17. TGA curve of **4** in nitrogen atmosphere. Note: a weight loss was observed from 280 °C to 290 °C due to the release of axial -COOH groups.

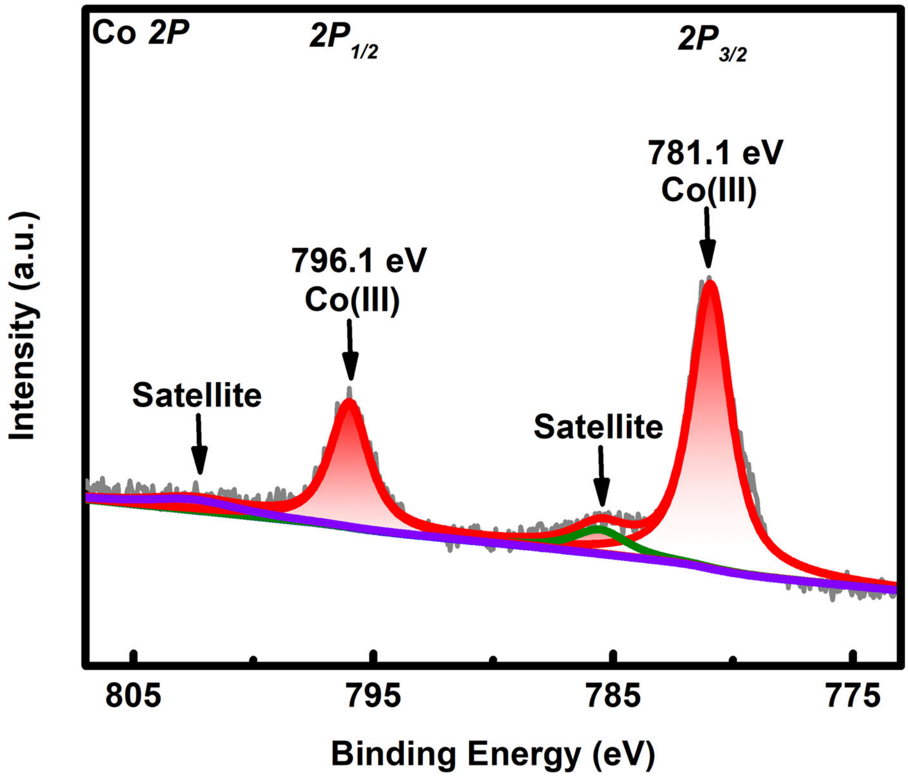

Supplementary Fig. 18. XPS measurement of **5**.

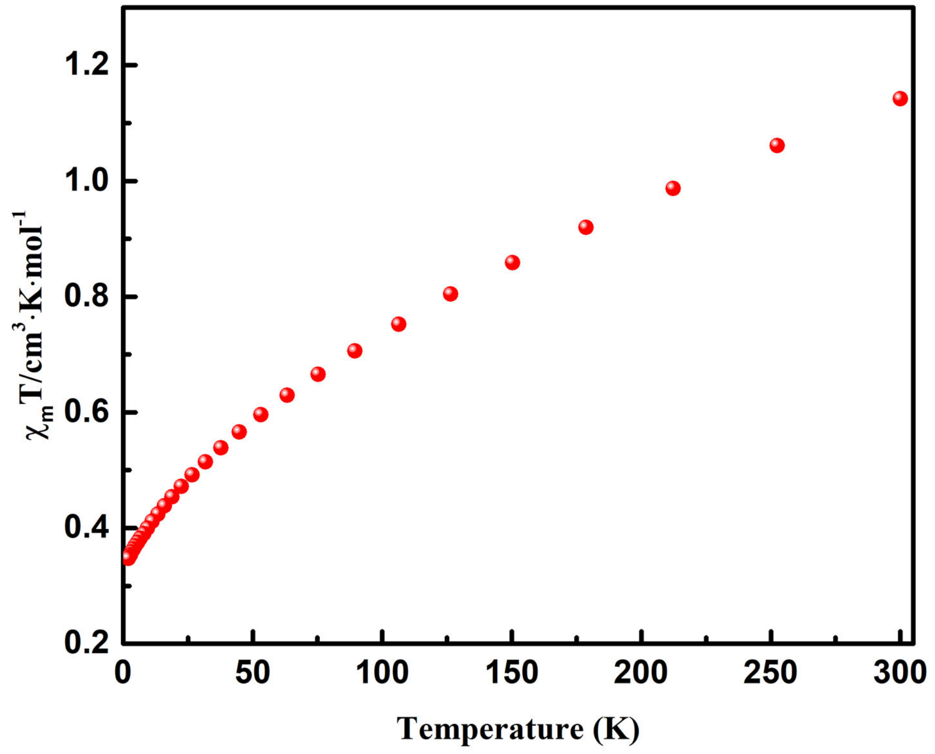

Supplementary Fig. 19. Plot of χ_MT vs T for **5**.

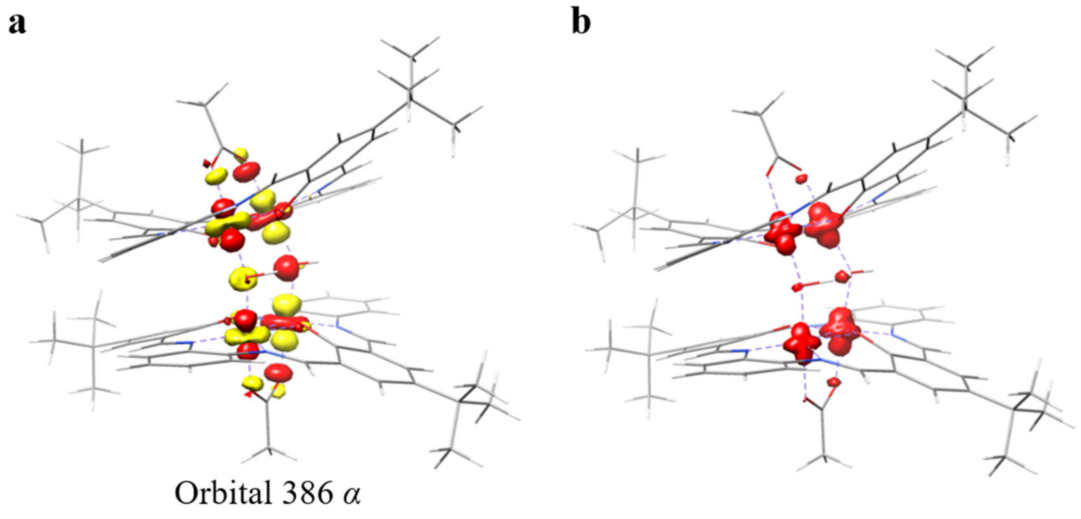

Supplementary Fig. 20. Unrestricted corresponding orbitals (with overlap coefficient equals 0) of **5** (a) and calculated spin density of **5** (b). Counteranions have been omitted for clarity.

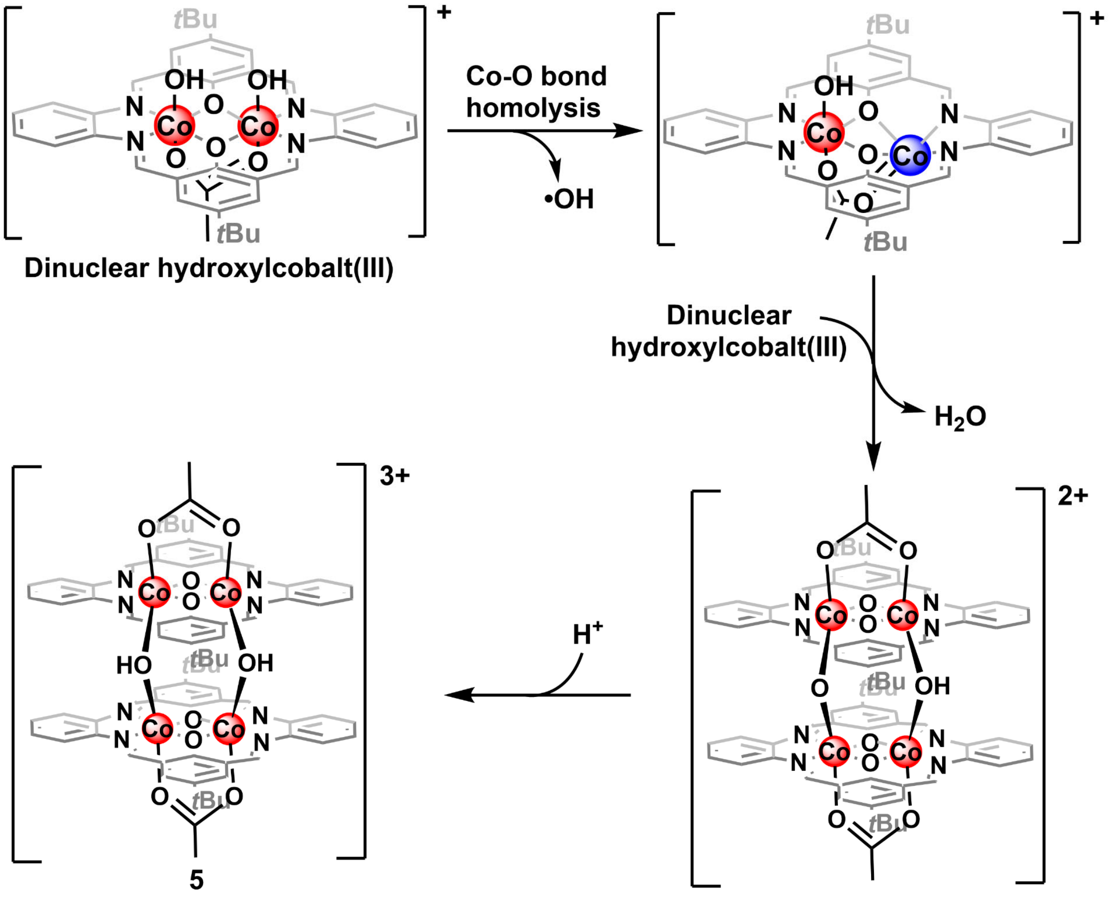

Supplementary Fig. 21. The plausible generation pathway of **5** from the proposed dinuclear hydroxylcobalt(III) intermediate. Counteranions have been omitted for clarity.

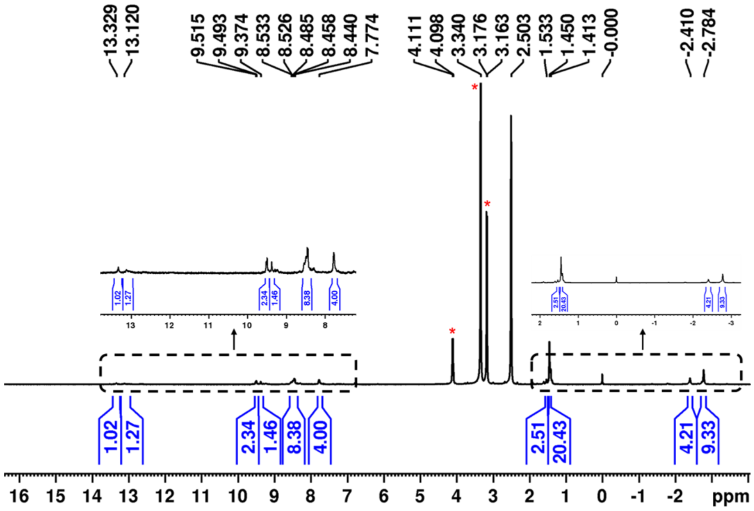

Supplementary Fig. 22. ^{1}H NMR spectrum of precipitates formed during the production of oxalic acid catalyzed by **4** in *d*$_6$-DMSO. (* denotes the resonance of MeOH).

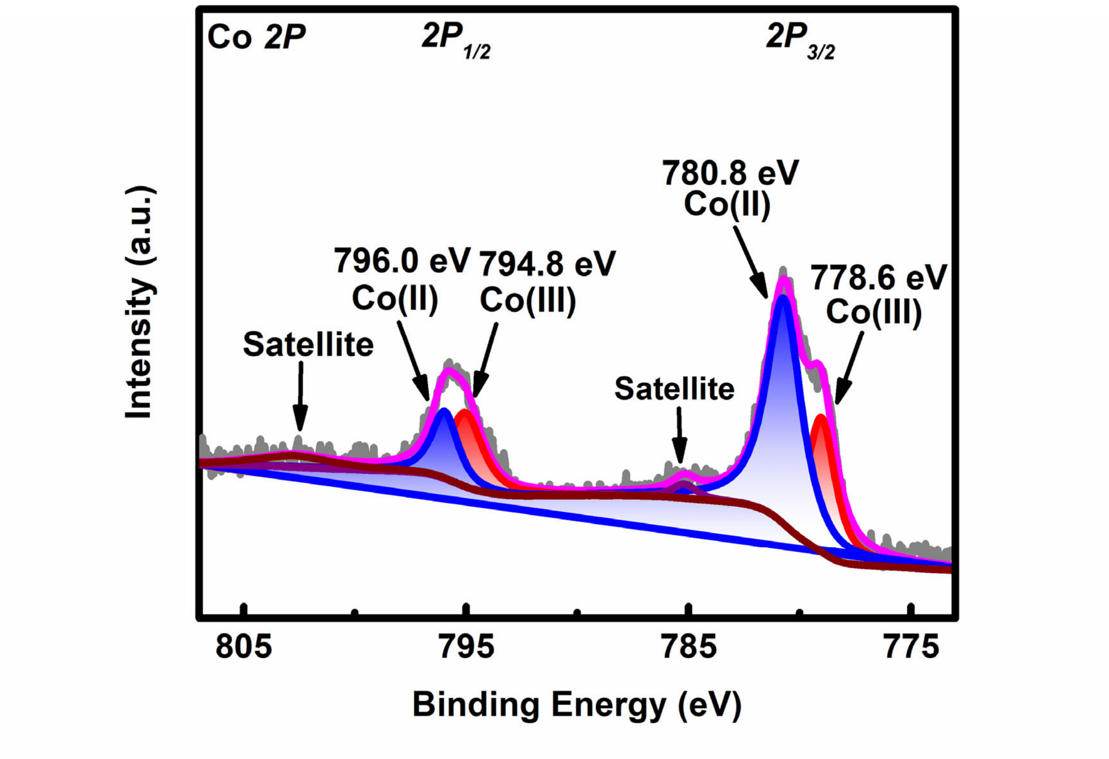

Supplementary Fig. 23. *XPS* measurement of the precipitates formed during the production of oxalic acid catalyzed by **4**.

4.0 mL of methanol solution containing 0.0170 mmol of complex **4** was transferred into a 25.0 mL Schlenk flask. After three freeze-pump-thaw cycles, 1 atm of O_2 was inflated into the Schlenk flask, followed by the addition of 1 atm of ^{13}CO. The Schlenk flask was set 20.0 cm aside a 500 W xenon lamp at 30 °C for 28 h. Then, excess amount of $CaCl_2$ was added to the reaction solution, and the resulting white precipitate was collected and used for IR analysis. Similar shifting trend has also been reported for unlabeled and ^{13}C-labeled oxalates.

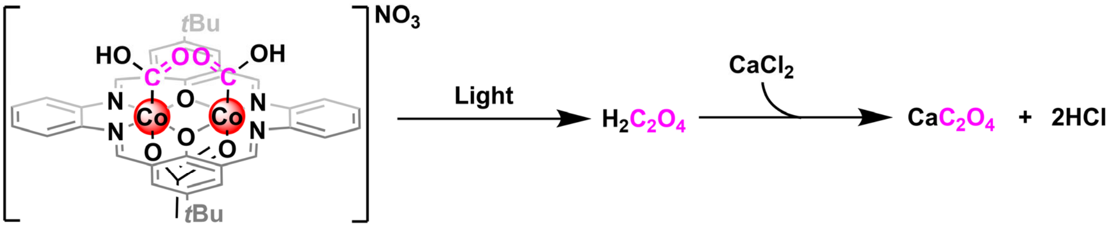

4

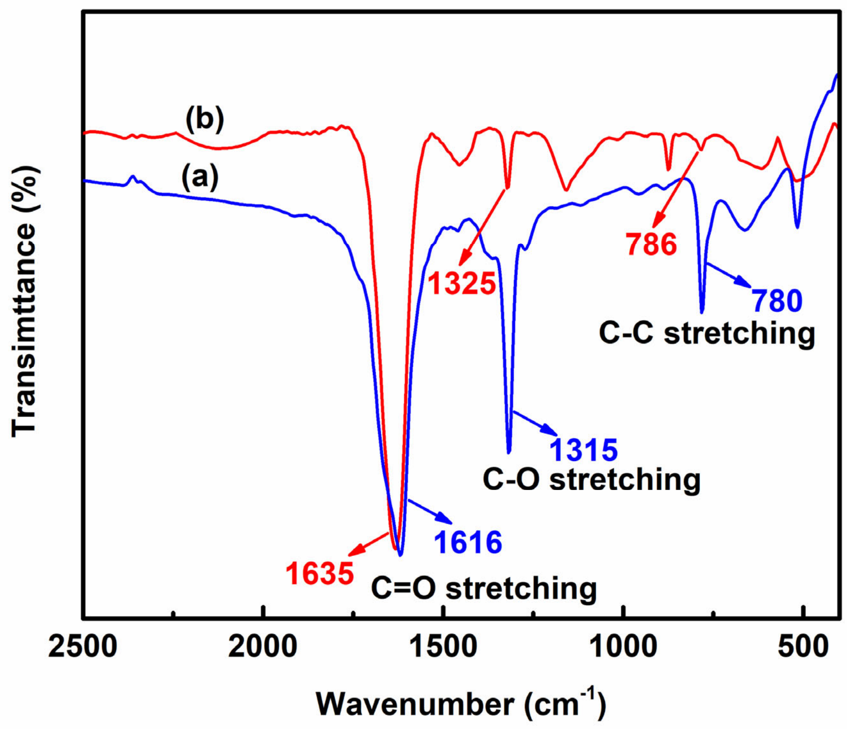

Supplementary Fig. 24. IR spectra of $Ca^{13}C_2O_4$ (a, obtained by adding $CaCl_2$ to the reaction solution of the catalytic production of oxalic acid from ^{13}CO) and $Ca^{12}C_2O_4$ (b).

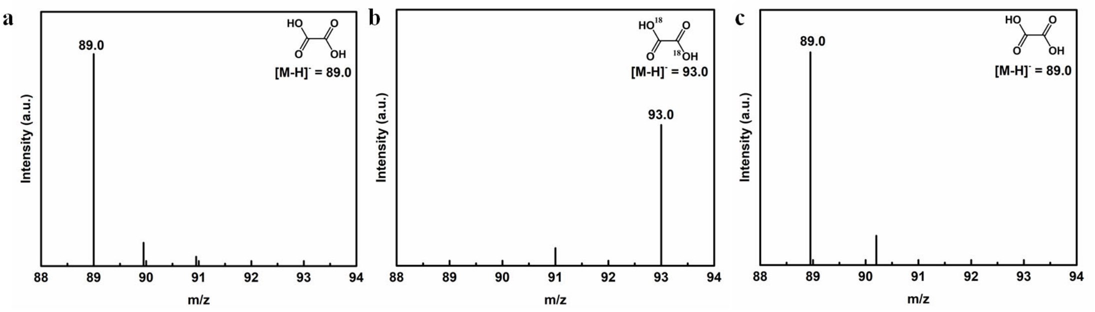

Supplementary Fig. 25. The MS measurements (negative mode) of $H_2C_2O_4$ standard reagent (a), and $H_2C_2O_4$ produced using $H_2{}^{18}O/{}^{16}O_2$ (b) and $H_2{}^{16}O/{}^{18}O_2$ (c).

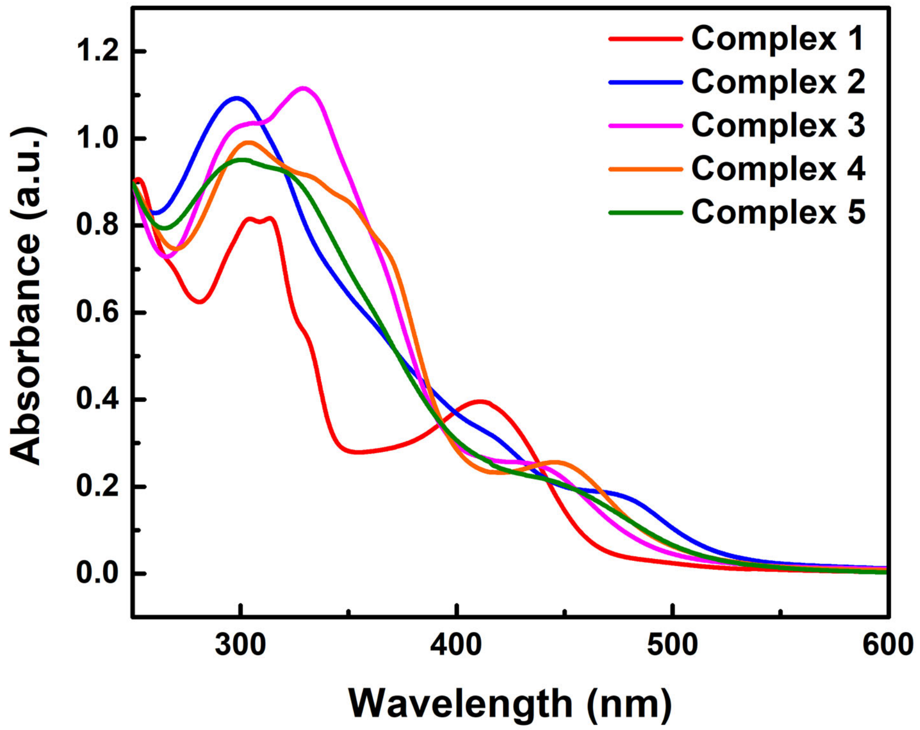

Supplementary Fig. 26. UV-Vis spectra of **1-5** in ethanol.

The formation of H_2O_2 in catalytic reaction was detected using the iodometry method[7]. The reaction solution was extracted with diethyl ether. The precipitate was filtered off and the filtrate was concentrated under reduced pressure. 3.0 mL of KI aqueous solution (0.17 M) was added to the concentrated solution and then filtered by Nylon membrane. The filtrate was used for the UV-Vis measurement.

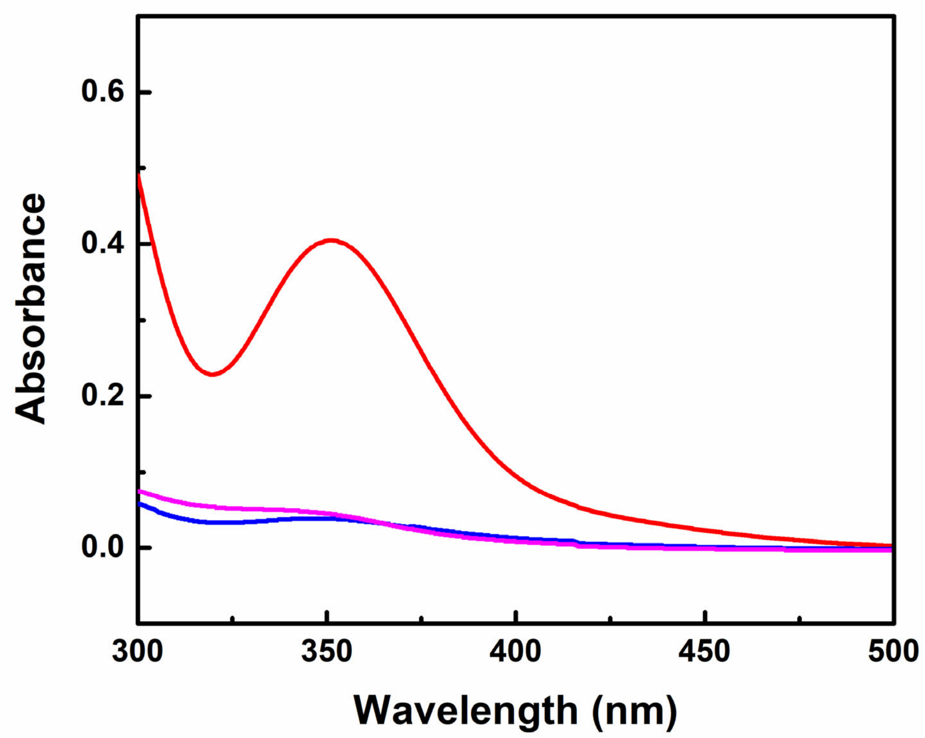

Supplementary Fig. 27. Detection of H_2O_2 generated in the production of oxalic acid catalyzed by **3** (red), **4** (blue) and **5** (magenta) using iodometry method.

(i) Neocuproine/$CuSO_4$ titration based colorimetric method[8]. The reaction solution was extracted with diethyl ether. The precipitate was filtered off and the filtrate was concentrated under reduced pressure. 2.0 mL of the colorimetric titrant (6 mM neocuproine, 4.2 mM $CuSO_4$, 25/75 (v/v) ethanol/DI water mixture) was added to the concentrated solution and then filtered by Nylon membrane. The UV-Vis spectrum of filtrate was then recorded. The absorbance at 454 nm was used to determine the H_2O_2 concentration based on the calibration curve.

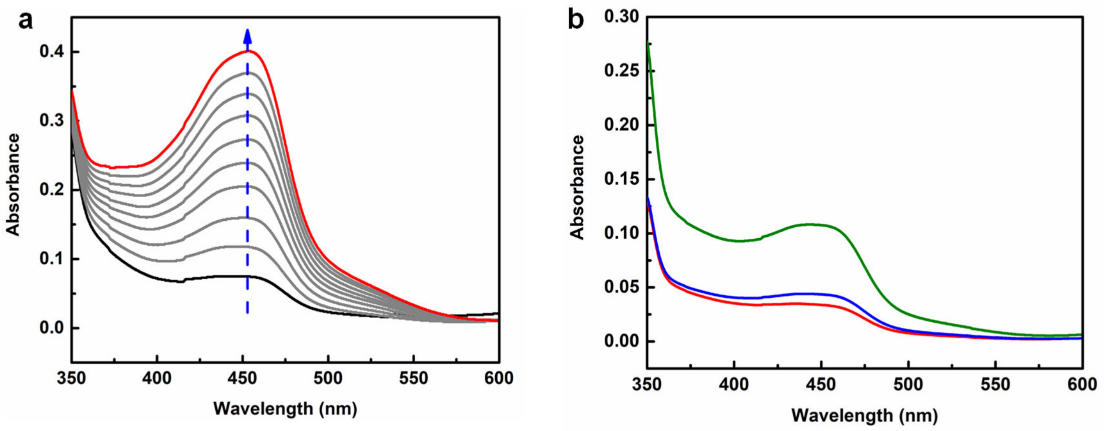

Supplementary Fig. 28. H_2O_2 detection based on neocuproine/$CuSO_4$ titration: (a) UV-Vis spectra recorded for the titration of H_2O_2 standard samples. Arrow indicates the change of the absorption intensity with different [H_2O_2] (0, 0.004, 0.008, 0.012, 0.016, 0.020, 0.024, 0.028, 0.032, and 0.036 mM); (b) UV-Vis spectra recorded for the titrations of H_2O_2 formed in the production of oxalic acid catalyzed by **3** (red), **4** (blue) and **5** (olive).

(ii) Cerium sulfate titration based colorimetric method[9] ($2\ Ce^{4+} + H_2O_2 \rightarrow 2\ Ce^{3+} + 2\ H^+ + O_2$). The reaction solution was extracted with diethyl ether. The precipitate was filtered off and the filtrate was concentrated under reduced pressure. 2.0 mL of the colorimetric titrant (Methanol solution of cerium sulfate, 6 mM) was added to the concentrated solution and then filtered by Nylon membrane. The filtrate was then analyzed by UV-Vis spectrometer. The amount of H_2O_2 can be calculated as half of the consumed Ce^{4+} ($1\ Ce^{4+} \approx 1/2\ H_2O_2$). The concentrations of Ce^{4+} before and after the reaction were determined by UV-Vis spectrometer at a wavelength of 316 nm.

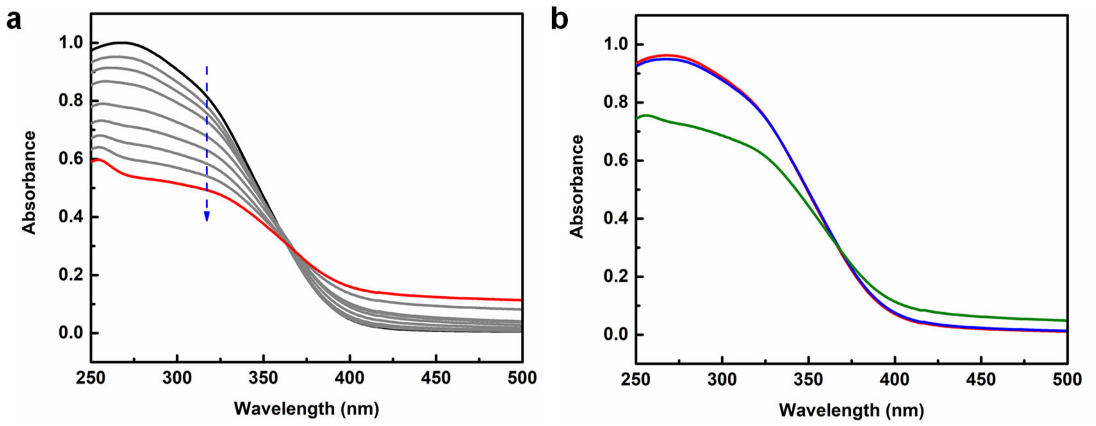

Supplementary Fig. 29. H_2O_2 detection based on Cerium sulfate titration: (a) UV-Vis spectra recorded for the titration of H_2O_2 standard samples. Arrows indicate the change of the absorption intensity with different [H_2O_2] (0, 0.004, 0.008, 0.012, 0.016, 0.020, 0.024, 0.028, and 0.032 mM); (b) UV-Vis spectra recorded for the titrations of H_2O_2 formed in the production of oxalic acid catalyzed by **3** (red), **4** (blue) and **5** (olive).

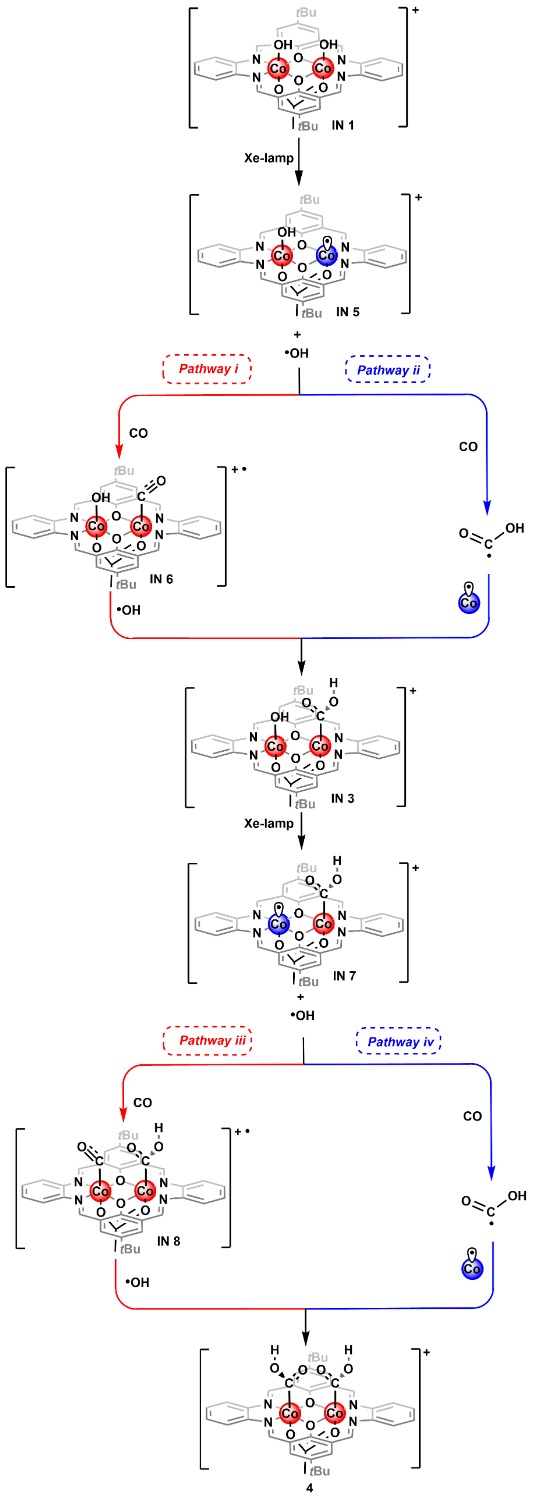

Supplementary Fig. 30. Plausible light-promoted pathways for the formation of **4** from dinuclear hydroxocobalt(III) complex. Counteranions have been omitted for clarity.

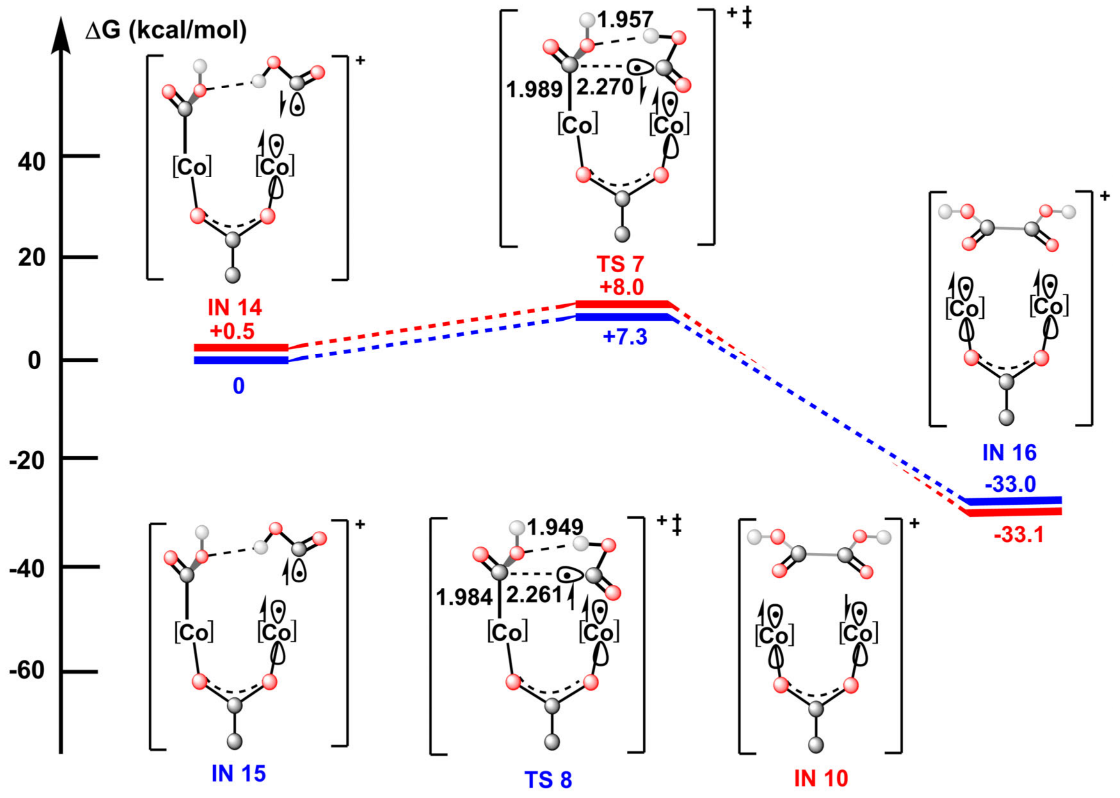

Supplementary Fig. 31. Gibbs free energy profiles for the generations of oxalic acid from **4** via the hydroxycarbonyl radical attack based pathway (bond lengths and distances are provided in Å). Counteranions have been omitted for clarity.

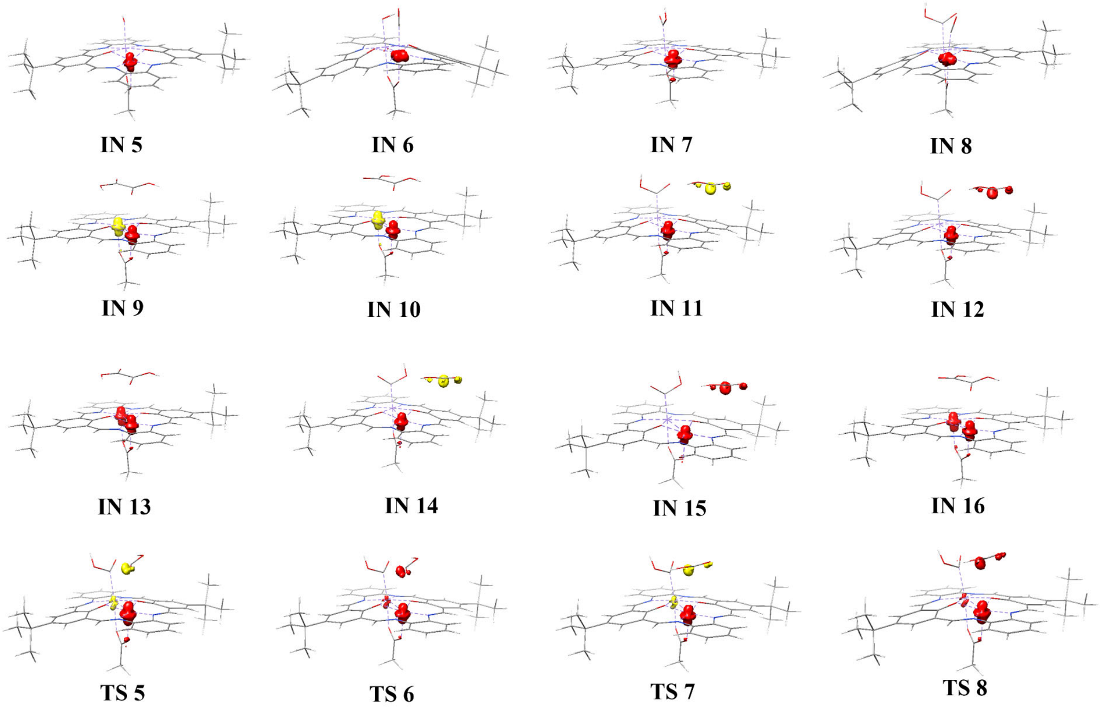

Supplementary Fig. 32. Spin density plots of **IN 5-16** and **TS 5-8**. Counteranions have been omitted for clarity.

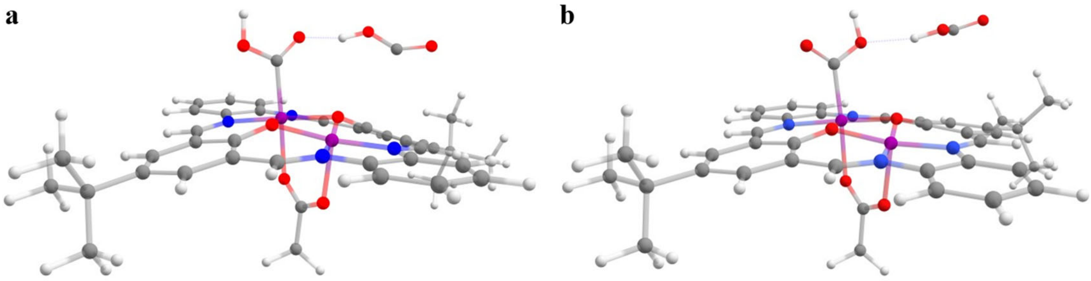

Supplementary Fig. 33. The structures of the minimal energy crossing points (MECPs) of **IN 11** / **IN 12** (a) and **IN 14** / **IN 15** (b). Counteranions have been omitted for clarity.

Crystallographic data was collected on Rigaku XtalAB Pro MM007 DW diffractometer with graphite monochromated Cu Kα radiation (= 1.54178 A). The diffractive measurement device type is 'XtaLAB AFC12 (RINC): Kappa single'. Structures were solved using direct method and then refined using SHELXL-2014 and Olex2[10-12] to convergence, in which all the non-hydrogen atoms were refined anisotropically during the final cycles. All hydrogen atoms of the organic molecule were placed by geometrical considerations and were added to the structure factor calculation. For **1**, **2**, **3** and **5**, we used the PLATON SQUEEZE procedure to remove the uncoordinated solvent molecules which could not be modeled properly[13]. Additionally, for **3**, we refined the structure by using some necessary restrains of anisotropy, such as RIGU and SADI for the counter cation fragments. For **4**, we refined the structure with the using of DELU command for the -COOH fragments. As a consequence of the packing forcing, the O7-H7 bond oriented to a direction that hampered the formation of a normal hydrogen bonding in **4**. Additionally, we refined the structure by using SIMU command for **5** and omitting the some of the most disagreeable points. As a consequence of the packing forcing, the O15-H15 bond oriented to a direction that hampered the formation of a normal hydrogen bonding in **5**.

Supplementary Table 1. Summary of crystallographic data collection and structure refinement for **1-5**

	**1**	**2**	**3**	**4**	**5**
Empirical formula	$C_{48}H_{62}Co_2N_4O_{10}$	$C_{84}H_{94}Co_4N_8O_{16}$	$C_{78}H_{82}CeCo_4N_{14}O_{30}$	$C_{42}H_{49}Co_2N_5O_{14}$	$C_{152}H_{160}CeCo_8N_{26}O_{54}$
Formula weight	972.88	885.74	2071.42	965.72	3749.52
Crystal system	triclinic	triclinic	triclinic	monoclinic	triclinic
Space group	P-1	P-1	P-1	P 21/n	P-1
T/K	100	100	100	100	100
a/Å	8.9620(4)	9.2874(4)	12.7711(2)	8.6478(1)	13.9916(4)
b/Å	9.3819(5)	16.0183(7)	13.5945(2)	29.4492(2)	17.4462(5)
c/Å	16.2138(6)	18.0697(7)	14.5111(2)	16.3632(1)	21.1759(5)
α/deg	74.788(4)	115.815(4)	76.569(1)	90	75.589(2)
β/deg	79.401(3)	104.125(3)	80.196(1)	94.499(1)	79.057(2)
γ/deg	62.145(5)	90.079(3)	89.077(1)	90	74.055(2)
V/Å^3	1160.07(11)	2328.86(19)	2413.96(6)	4154.39(6)	4772.7(2)
Z	1	2	1	4	1
D/g cm^{-3}	1.393	1.263	1.425	1.544	1.305
μ/mm^{-1}	6.111	5.988	9.524	6.906	7.695
F(000)	512.0	926.0	1054.0	2008.0	1917.9
Reflections collected	10257	19470	23217	23191	50290
Independent reflections	4689<br>R_{int} = 0.0335<br>R_{sigma} = 0.0396	9189<br>R_{int} = 0.0433<br>R_{sigma} =0.0596	9851<br>R_{int} = 0.0332<br>R_{sigma} = 0.0304	8663<br>R_{int} = 0.0326<br>R_{sigma} = 0.0377	16653<br>R_{int} = 0.0562<br>R_{sigma} = 0.0643
Data/restraints/parameters	4689/0/308	9189/0/516	9851/324/689	8663/4/582	16612/4368/1095
Goodness-of-fit on F^2	1.064	1.104	1.047	1.068	1.062
R_1/wR_2 indexes [I>2σ(I)]	R_1 = 0.0537<br>wR_2 = 0.1278	R_1 =0.0712<br>wR_2 = 0.2027	R_1 = 0.0595<br>wR_2 = 0.1673	R_1 = 0.0645<br>wR_2 = 0.1762	R_1 = 0.0957<br>wR_2 = 0.1898
R_1/wR_2 indexes [all data]	R_1 = 0.0489<br>wR_2 = 0.1306	R_1 = 0.0682<br>wR_2 = 0.1982	R_1 = 0.0579<br>wR_2 = 0.1690	R_1 = 0.0591<br>wR_2 = 0.1809	R_1 = 0.0678<br>wR_2 = 0.2080
CCDC	2209326	2209330	2209329	2209332	2209333

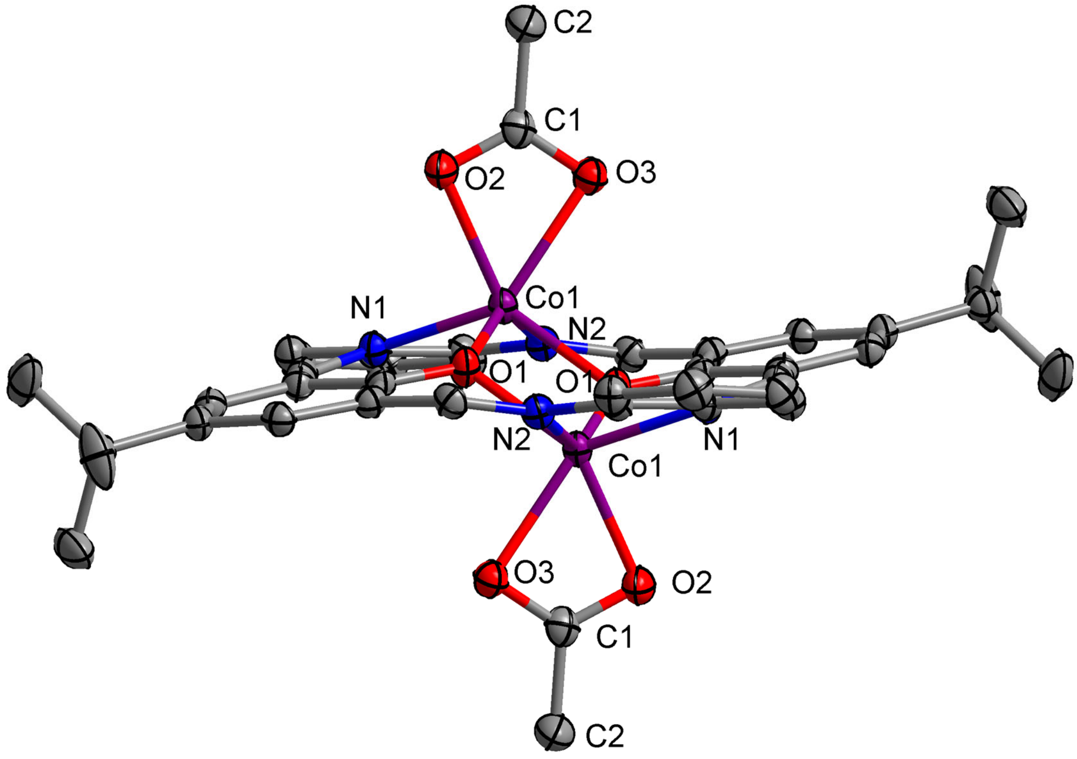

Supplementary Fig. 34. ORTEP representations (50% probability) of **1**. Solvent molecules and hydrogen atoms have been omitted for clarity.

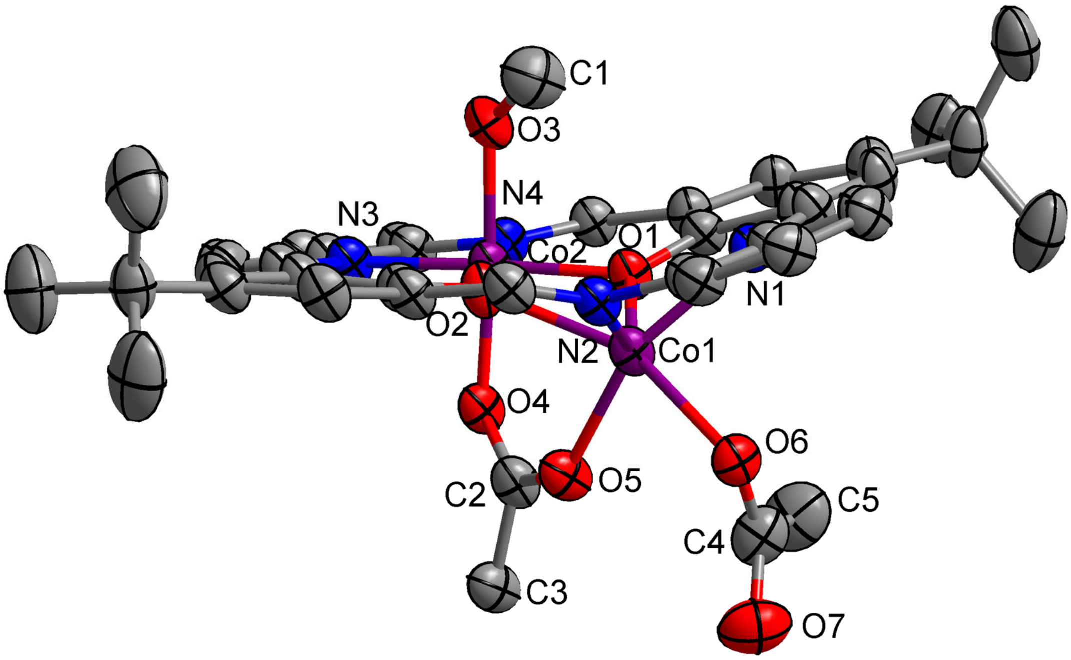

Supplementary Fig. 35. ORTEP representations (50% probability) of **2**. Solvent molecules and hydrogen atoms have been omitted for clarity.

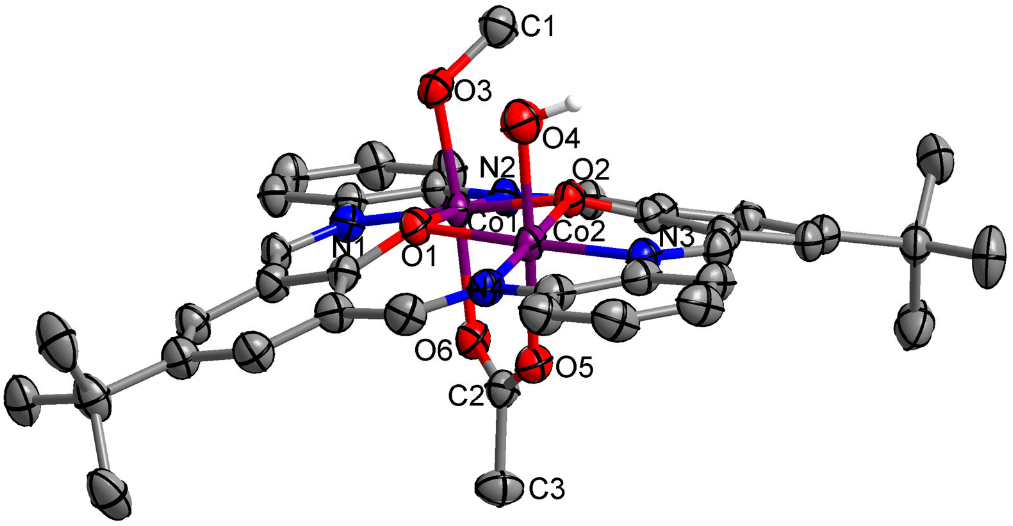

Supplementary Fig. 36. ORTEP representations (50% probability) of **3**. Solvent molecules, counteranions, and hydrogen atoms, except for those on oxygen atoms, have been omitted for clarity.

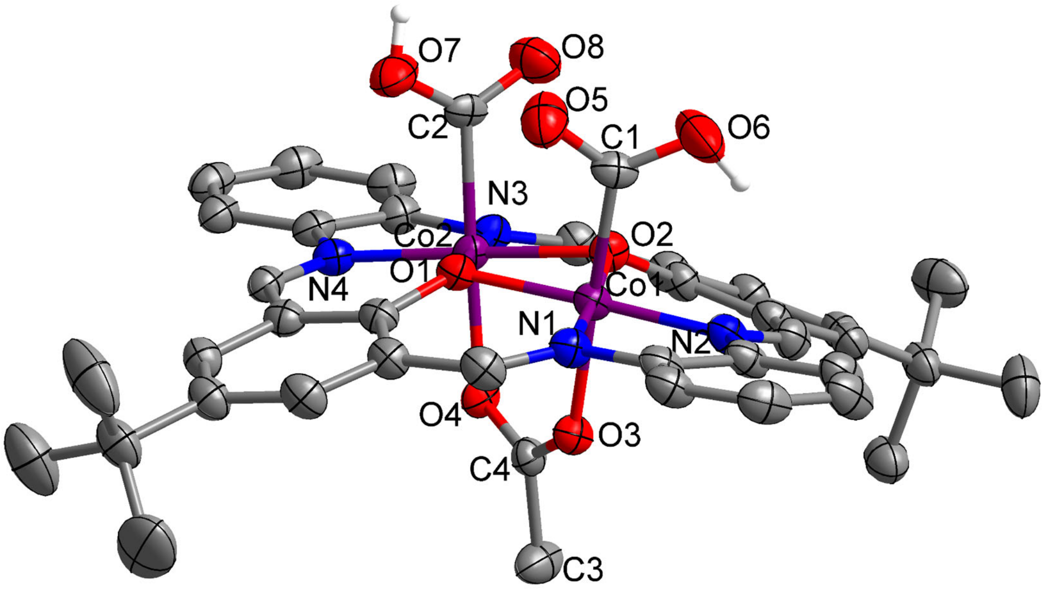

Supplementary Fig. 37. ORTEP representations (50% probability) of **4**. Solvent molecules, counteranions, and hydrogen atoms, except for those on oxygen atoms, have been omitted for clarity.

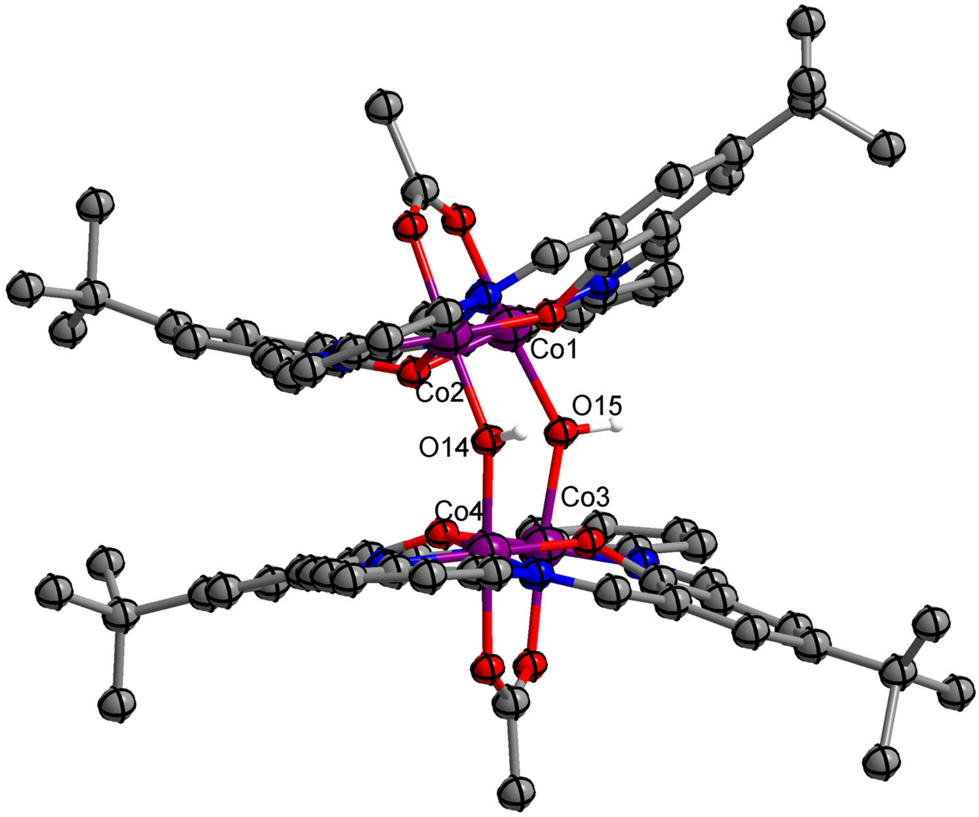

Supplementary Fig. 38. ORTEP representations (50% probability) of **5**. Solvent molecules, counteranions, and hydrogen atoms, except for those on oxygen atoms, have been omitted for clarity.

Supplementary Table 2. Selected bond lengths (Å) and angles (deg) of the solid state structures of **1-5**

	**1**	**2**	**3**	**4**	**5**
Co-$O_{equatorial}$ (Å)	1.989(1)/ 1.989(3)/ 2.027(2)/ 2.027(1)	2.063(2)/ 2.144(3)/ 1.881(3)/ 1.868(2)	1.914(2)/ 1.894(2)/ 1.896(2)/ 1.900(2)	1.897(2)/ 1.898(2)/ 1.889(2)/ 1.904(2)	1.918(3)/ 1.897(3)/ 1.905(4)/ 1.890(3)/ 1.890(4)/ 1.890(4)/ 1.883(4)/ 1.909(4)
Co-$N_{equatorial}$ (Å)	2.057(2)/ 2.057(2)/ 2.077(2)/ 2.077(2)	2.108(3)/ 2.071(3)/ 1.867(3)/ 1.870(3)	1.859(3)/ 1.874(3)/ 1.868(3)/ 1.866(3)	1.870(3)/ 1.876(3)/ 1.871(3)/ 1.875(3)	1.863(5)/ 1.872(4)/ 1.871(4)/ 1.874(4)/ 1.871(4)/ 1.861(4)/ 1.861(5)/ 1.883(5)
Co-O_{AcO} (Å)	2.164(2)/ 2.164(2)/ 2.211(2)/ 2.211(2)	2.076(3)/ 2.045(3)/ 1.941(3)/	1.954(2)/ 1.919(2)	1.936(2)/ 1.969(2)	1.926(4)/ 1.907(4)/ 1.938(4)/ 1.894(4)
Co-O_{MeO} (Å)		1.884(3)	1.871(3)		
Co-C_{COOH} (Å)	-	-	-	1.918(3)/ 1.913(3)	-

Co-$O_{\mu\text{-}OH}$ (Å)	-	-	-	-	1.905(4)/<br>1.909(4)/<br>1.907(4)/<br>1.895(4)
Co-O_{OH} (Å)	-	-	1.935(3)	-	-
C=O (Å)	-	-	-	1.193(5)/<br>1.207(4)	-
C(O)-OH (Å)	-	-	-	1.231(5)/<br>1.249(4)	-
O-C-O_{COOH} (deg)	-	-	-	120.5(3)/<br>122.1(3)	-

Supplementary Table 3. Comparisons of the RMSDs of the selected bonds (labelled in blue) and calculated C=O stretching frequencies of **4** using different density functionals[a].

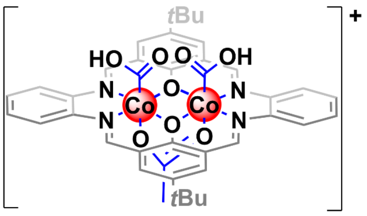

Functionals	IR/ cm^{-1}	RMSD[b]/ Å
Exp.	1697/1670	-
B97-3c	1724/1685	0.0325
B3LYP	1739/1704	0.0410
BP	1687/1646	0.0412
TPSS	1689/1649	0.0367
M06	1784/1756	0.0303
PBE0	1775/1743	0.0268
M06L	1756/1719	0.0375

[a] The hybrid B3LYP/M06/PBE0 functionals and GGA type TPSS/M06L/BP functional were used for geometry optimizations, in combination with triple-ζ quality def2-mTZVP basis sets for all elements. The resolution of the identity plus chain of spheres approximation[14] (RIJCOSX, for B3LYP/M06/PBE0) and the resolution of the identity approximation[15-16] (RI, for TPSS/M06L/BP) were used to accelerate the calculations with the auxiliary basis set def2/J; [b] the RMSDs were calculated for the selected bond lengths referring to the values of the crystal structure of complex **4**.

References

(1) Swamy, P. C.; Solel, E.; Reany, O.; Keinan, E. Synthetic Evolution of the Multifarene Cavity from Planar Predecessors. *Chem. Eur. J.* **24**, 15319-15328 (2018).

(2) Brandenburg, J. G.; Bannwarth, C.; Hansen, A.; Grimme, S. *J. Chem. Phys*. **148**, 064104 (2018).

(3) Chan, B., Dawson, W. & Nakajima, T. Searching for a reliable density functional for molecule-environment interactions, found B97M-V/def2-mTZVP. *J. Phys. Chem. A*. **126**, 2397−2406 (2022).

(4) Barone, V.; Cossi, M. Quantum calculation of molecular energies and energy gradients in solution by a conductor solvent model. *J. Phys. Chem. A* **102**, 1995−2001 (1998).

(5) Swamy, P. C.; Solel, E.; Reany, O.; Keinan, E. Synthetic Evolution of the Multifarene Cavity from Planar Predecessors. *Chem. Eur. J.* **24**, 15319-15328 (2018).

(6) Xu, R., Chakraborty, S., Yuan, H. & Jones, W. D. Acceptorless, Reversible dehydrogenation and hydrogenation of N-heterocycles with a cobalt pincer catalyst. *ACS Catal*. **5**, 6350-6354 (2015).

(7) Jung, O. et al. Highly active NiO photocathodes for H_2O_2 production enabled via outer-sphere electron transfer. *J. Am. Chem. Soc.,* **140**, 4079−4084(2018).

(8) Huang, A.X. et al. Direct H_2O_2 synthesis, without H_2 gas. *J. Am. Chem. Soc.*, **144**, 14548−14554 (2022).

(9) Yu, Z.Y. et al. Low-coordinated Pd site within amorphous palladium selenide for active, selective, and stable H_2O_2 electrosynthesis. *Adv. Mater*., **35**, 2208101 (2023).

(10) Sheldrick, G. M. SHELXT – Integrated space-group and crystal-structure determination. *Acta Cryst. A*. **71**, 3–8 (2015).

(11) Dolomanov, O. V. et al. OLEX2: a complete structure solution, refinement and analysis program. *J. Appl. Crystallogr*. **42**, 339–341 (2009).

(12) Spek, A. L. Structure validation in chemical crystallography. *Acta Cryst. D*. **65**, 148–155 (2009).

(13) Spek, A. L. PLATON SQUEEZE: a tool for the calculation of the disordered solvent contribution to the calculated structure factors. *Acta Crystallogr. Sect.C. Cryst. Struct. Commun*. **71**, 9–18 (2015).

(14) Neese, F.; Wennmohs, F.; Hansen, A.; Becker, U. Efficient, approximate and parallel Hartree–Fock and hybrid DFT calculations. A 'chain-of-spheres' algorithm for the Hartree–Fock exchange. *Chem. Phys*. **356**, 98 (2009).

(15) Kendall, R. A.; Früchtl, H. A. The impact of the resolution of the identity approximate integral method on modern ab initio algorithm development. *Theor. Chem. Acc.* **97**, 158 (1997).

(16) Eichkorn, K.; Weigend, F.; Treutler, O.; Ahlrichs, R. Auxiliary basis sets for main row atoms and transition metals and their use to approximate Coulomb potentials. *Theor. Chem. Acc*. **97**, 119 (1997).
