# Supplementary material for: Direct synthesis of oxalic acid via oxidative CO coupling mediated by a dinuclear hydroxycarbonylcobalt(III) complex
Source: Nat Commun. 2023 May 12;14:2739. doi: 10.1038/s41467-023-38442-4 (PMC10182058; doi:10.1038/s41467-023-38442-4)
Supplement: Supplementary file 3 — Description of Additional Supplementary Files [file 41467_2023_38442_MOESM3_ESM.pdf]

Description of Additional Supplementary Files

File Name: Supplementary Data 1
Description: Crystallographic Data of Complexes 1-5

File Name: Supplementary Data 2
Description: Coordinates of Optimized Structures Used in Computational Studies
